# Hybrid integrated photonics using bulk acoustic resonators

**DOI:** 10.1038/s41467-020-16812-6

**Published:** 2020-06-17

**Authors:** Hao Tian, Junqiu Liu, Bin Dong, J. Connor Skehan, Michael Zervas, Tobias J. Kippenberg, Sunil A. Bhave

**Affiliations:** 1 OxideMEMS Lab, Purdue University, 47907 West Lafayette, IN USA; 2 Institute of Physics, Swiss Federal Institute of Technology Lausanne (EPFL), 1015 Lausanne, Switzerland

**Keywords:** Nanophotonics and plasmonics, Integrated optics, Microresonators, Photoacoustics

## Abstract

Integrated photonic devices based on Si_3_N_4_ waveguides allow for the exploitation of nonlinear frequency conversion, exhibit low propagation loss, and have led to advances in compact atomic clocks, ultrafast ranging, and spectroscopy. Yet, the lack of Pockels effect presents a major challenge to achieve high-speed modulation of Si_3_N_4_. Here, microwave-frequency acousto-optic modulation is realized by exciting high-overtone bulk acoustic wave resonances (HBAR) in the photonic stack. Although HBAR is ubiquitously used in modern communication and superconducting circuits, this is the first time it has been incorporated on a photonic integrated chip. The tight vertical acoustic confinement releases the lateral design of freedom, and enables negligible cross-talk and preserving low optical loss. This hybrid HBAR nanophotonic platform can find immediate applications in topological photonics with synthetic dimensions, compact opto-electronic oscillators, and microwave-to-optical converters. As an application, a Si_3_N_4_-based optical isolator is demonstrated by spatiotemporal modulation, with over 17 dB isolation achieved.

## Abstract (web summary)

Here, the authors demonstrate acousto-optic modulation of silicon nitride microring resonators using high-overtone bulk acoustic wave resonances, allowing modulation in the GHz range via acoustic waves. As an application, an optical isolator is demonstrated with 17 dB non-reciprocity.

## Introduction

Integrated photonics has drawn increasing attention in recent years. Although within the last decade, silicon photonics has transitioned from laboratory-based research into commercial products used in data centers, major efforts are still underway with regards to silicon nitride (Si_3_N_4_) photonic devices^1^. The Si_3_N_4_ platform has attracted intense efforts due to its wide transparency window from the visible to mid-infrared, ultralow linear losses^1^, absence of two-photon absorption in the telecommunication band, large Kerr nonlinearity *χ*^(3)^, small Brillouin gain^2^, and flexibility for waveguide geometric dispersion engineering^3^. Silicon nitride has been a popular material of choice for dissipative Kerr soliton microcomb generation^4^. In addition, supercontinuum generation^3^, optical filters^5^, gyroscopes^6^, and coherent telecommunication^7^ have been demonstrated using Si_3_N_4_. Recent advances of Si_3_N_4_-based soliton microcombs have included octave-spanning comb spectra^8,9^, ultralow initiation power^10–12^, and microcomb repetition rates in the microwave domain^13^. Reliable and fast resonance tuning of Si_3_N_4_ microresonators is an important asset and requirement for multiple applications in integrated photonics. For example, high-bandwidth tuning allows microcomb repetition rate stabilization^14^, or resonance tuning for filters^5^. Likewise, recently emerged platforms such as spatiotemporal-modulation-based optical nonreciprocity^15^ and topological optical bandstructures^16,17^ require GHz-rate modulation within optical microresonators, which poses stringent requirements on the cross-talk and size. However, due to the inversion symmetry, and thus the lack of *χ*^(2)^ nonlinearity, it is challenging to electrically modulate the refractive index of Si_3_N_4_. Traditionally, the thermo-optical effect is employed to fulfill the tuning requirement^18,19^. However, thermal tuning presents low tuning speed (~1 kHz), high power consumption (~10 mW), and large cross-talk. These drawbacks make it incompatible with large-scale integration and cryogenic applications^20^. Although hybrid integration with various electro-optical materials, e.g., graphene^21^, lead zirconate titanate (PZT)^22^, lithium niobate (LiNbO_3_)^23^, and monolayer WS_2_^24^, has made significant progress, there are still remaining challenges related to Complementary Metal Oxide Semiconductor (CMOS) compatibility, fabrication complexity, optical loss, and dispersion engineering. To fully utilize the maturity and advantages of Si_3_N_4_ photonics, new tuning mechanisms that retain the original optical properties (e.g., waveguide low-loss and dispersion) are needed.

**Fig. 1:**
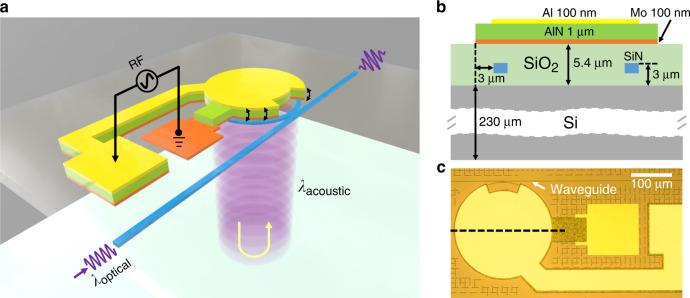
Hybrid nanophotonic high-overtone bulk acoustic resonator (HBAR) platform. **a** 3D schematic illustrating excitation of bulk acoustic waves via a macroscopic piezoelectric actuator, which transmit vertically into the stack and form acoustic standing waves inside the acoustic Fabry–Pérot cavity. The resonance-enhanced mechanical stress changes the waveguide’s refractive index via the stress-optical effect and thereby modulates the output optical intensity. **b** Cross-section of the device along the black dashed line in **c**, with critical dimensions labeled. The AlN piezoelectric actuator is placed directly on top of the Si_3_N_4_ microring resonator which is embedded in 5.4 μm of SiO_2_ cladding. **c** Optical microscope image of the fabricated device.

The stress-optical effect, discovered over a 100 years ago, has recently gained attention for its role in the modulation of Si_3_N_4_ waveguides and microring resonators, both theoretically^25,26^ and experimentally^27,28^, thanks to the advances in Micro-Electro-Mechanical Systems^29^. In this work, by integrating aluminium nitride (AlN) piezoelectric actuators on top of Si_3_N_4_ photonic devices, we demonstrate, to the best of our knowledge, the first acousto-optic modulation (AOM) of Si_3_N_4_ microring resonators using high-overtone bulk acoustic wave resonances (HBARs)^30^. Sub-micrometer wavelength acoustic waves are excited by macroscopic actuators, which transmit vertically into the substrate and perpendicular to optical paths (see Fig. 1a). Trapped inside a Fabry–Pérot-like acoustic cavity that is formed by the top and bottom surfaces of the entire substrate, a rich family of acoustic resonant modes is efficiently excited at the microwave frequencies up to 6 GHz. The coupling between vertical acoustic waves and in-plane optical circuits makes it possible for independent optimization of the actuator and optical components. Specifically, Si_3_N_4_ waveguide can be buried deeply inside the oxide cladding for preserving the low optical losses. The high lateral acoustic mode confinement further enables low cross-talk and compact integration. These features are in stark contrast with conventional surface acoustic wave (SAW) AOM^26,31^, which requires an inter-digital transducer (IDT) with sub-micrometer electrode fingers, coplanar integration of IDT and optical waveguides, and perfect termination of acoustic waves to reduce cross-talk.

The same structure platform is also capable of linear bidirectional tuning of Si_3_N_4_ microring resonators (−0.2 pm V^−1^), which shows a high power efficiency of 5 nW pm^−1^ resonant wavelength tuning (in stark contrast to thermal tuning on the order of 1 mW pm^−1^^18^). Although similar optical resonance tuning can be achieved via PZT with 26 pm V^−1^ tuning ability^28^, AlN has the advantages of high actuation linearity, no hysteresis, high power handling (breakdown field  > 150 V μm^−1^), and low current leakage, which are more important for microcomb tuning and stabilization. For example, resonance tuning of 10 pm (corresponding to frequency excursion of GHz) is sufficient to control the soliton state^19^, which is within the reach of our ability. However, once the soliton state is accessed, the resonance needs to be anchored by the resonant actuator. In this case, the power consumption is the most important figure of merit to consider. The current demonstration of PZT tuning shows power consumption of 1.2 μW pm^−1^ due to its large leaky current^28^, which is two orders of magnitude larger. Moreover, the large loss-tangent of PZT makes it resistive at microwave frequencies, which prevents GHz modulation.

Here we show the GHz AOM of Si_3_N_4_ microring resonator by piezoelectrically exciting a family of the fundamental HBAR modes with free spectral range (FSR) of 17.5 MHz. The cross-talk between adjacent actuators is kept around −60 dB over wide frequencies. The tuning speed is fully explored, demonstrating sub-nanosecond actuation capability, which is mainly limited by intrinsic acoustic resonances. This simple but multi-functional stress-optical platform demonstrated in this work would enable advanced applications in Si_3_N_4_ photonic platform^32^, such as the repetition rate stabilization of soliton microcombs via injection locking^33^, on-chip optomechanical frequency comb generation^34^, and comb-assisted microwave photonic filters^35^. As an application demonstration, we show in the last section the realization of a fully integrated, electrically driven Si_3_N_4_ microring isolator via spatiotemporal modulation. A 17 dB isolation is achieved with 1 GHz bandwidth.

## Results

### Device design

The c-axis oriented polycrystalline AlN, with a piezoelectric coefficient *e*_33_ ~ 1.55 C m^−2^^36^, is utilized to form the piezoelectric actuator, which will be deformed and generate stress around the Si_3_N_4_ waveguide when a vertical electric field is applied. As shown in Fig. 1a, a disk-shaped actuator is placed directly on top of the Si_3_N_4_ microring resonator such that their edges are congrunt. When the actuator is driven harmonically, vibration of the AlN disk launches an acoustic wave vertically into the substrate (Fig. 1a). As the bottom surface of the substrate is smooth and flat, the acoustic wave is reflected and subsequently bounces back and forth between the top and bottom surfaces. Working as an acoustic Fabry–Pérot cavity, the counter-propagating acoustic waves will constructively interfere when the cavity length is an integer number of the acoustic wavelength, with acoustic energy trapped inside the cavity. These bulk acoustic standing waves further enhance the stress field around the Si_3_N_4_ waveguides and modulate the refractive index through stress-optical effect^25^.

The device cross-section is illustrated in Fig. 1b. To apply the vertical electric field, an AlN film of 1 μm thickness is sandwiched between top Al (100 nm thickness) and bottom Mo (100 nm thickness) metal layers. The Si_3_N_4_ waveguides are fabricated using a subtractive process^37^ and have a height of 0.8 μm and width of 1.8 μm. The waveguides are fully buried in SiO_2_ cladding of 5.4 μm thickness (2.4 μm SiO_2_ from the Mo layer to prevent metal absorption). The entire device sits on a 230 μm-thick Si substrate. The radius of the microring resonator is 118 μm, and is placed 3 μm within the edge of disk actuator. The final fabricated device is shown in Fig. 1c (see Methods for more fabrication details). The area around the waveguide-to-microring coupling region is opened to prevent any actuation-induced perturbation of light coupling between bus waveguide and microring resonator.

### High overtone bulk acoustic wave resonances

**Fig. 2:**
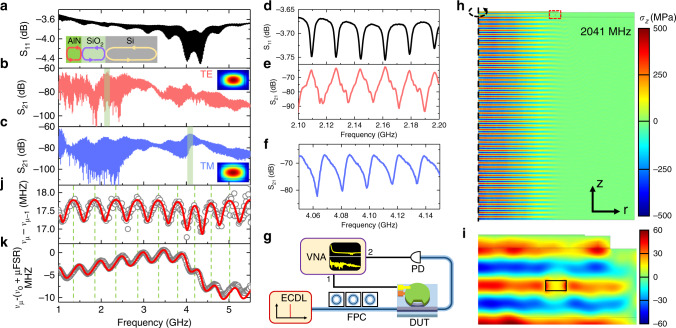
Microwave frequency electro-acousto-optic modulation. **a** Electromechanical S_11_ spectrum from 1 to 5.5 GHz. A range of equidistant bulk acoustic resonances is found to exist over a broad frequency range. The inset schematic illustrates the acoustic wave reflection at interfaces. **b**, **c** Optomechanical S_21_ responses of TE and TM modes demonstrate acousto-optic modulation covering multiple octave-spanning microwave frequencies. Due to different optical mode shapes (insets in **b** and **c**) and thus acousto-optic mode overlap, TE and TM modes show dissimilar S_21_ spectra. **d**, **e** The zoom-in of S_11_ and TE mode’s S_21_ within the window (green shaded area in **b**) around 2 GHz. **f** The zoom-in of TM mode’s S_21_ around 4 GHz in **c**. The resonances distribute evenly with an FSR of 17.5  MHz. **g** Schematic of the setup for measuring electromechanical and optomechanical response. DUT device under test, ECDL external cavity diode laser, FPC fiber polarization controller, PD photo-diode, VNA vector network analyzer. **h** Numerical simulation of vertical stress *σ*_*z*_ distribution for one typical acoustic resonant mode at 2.041 GHz under 1 V driving field, with a zoom-in around the optical waveguide (red box in **h**) shown in **i**. At several GHz, the acoustic wavelength is similar in scale to optical wavelength and waveguide structure. **j** The measured (circle) and calculated (solid line) frequency difference between each pair of adjacent Si resonances, showing a periodic variation of local FSR around an average value of 17.5 MHz. The maxima of FSR align with the SiO_2_ resonances marked by green dashed lines. **k** Measured (circle) and calculated (solid line) higher order dispersion represented by the frequency deviation from an equidistant frequency grid (with average FSR = 17.515 MHz), referencing to mode *ν*_0_ (3.0145 GHz). *μ* is the mode number difference relative to the mode at 3.0145 GHz.

The electromechanical reflection parameter S_11_ is first measured experimentally using port 1 of a network analyzer (Fig. 2g), which characterizes the energy conversion from electrical to mechanical vibration as a dip. As shown in Fig. 2a, a series of resonance dips is evenly distributed over multiple octaves in the microwave regime. A zoom-in of the spectrum is illustrated in Fig. 2d, highlighting the resonance shape, linewidth (~3 MHz), and acoustic FSR of around 17.5 MHz. The narrow linewidth demonstrates high mechanical *Q* (~1000), which is mainly limited by the intrinsic acoustic loss in the substrate and scattering at material interfaces. Meanwhile, the envelope of these sharp resonances varies slowly and smoothly with a period of  ~490 MHz. This is caused by the resonance inside the 5.4 μm SiO_2_ cladding layer, which stems from acoustic wave reflections at the Si-SiO_2_ interface due to an acoustic impedance mismatch. The position of SiO_2_ resonance is located at the node of the envelope, whereas anti-resonance is located at the anti-node. Intuitively speaking, at the anti-resonance of the SiO_2_ layer, it works effectively as an acoustic anti-reflection coating such that more acoustic energy will transmit into the Si substrate, which has larger acoustic impedance, and thus better electromechanical conversion. When the acoustic half wavelength matches the 1 μm AlN thickness, the AlN layer reaches its fundamental resonance mode and becomes more efficient in exciting acoustic waves around 4–4.3 GHz. It is noteworthy that, by optimizing AlN and SiO_2_ thicknesses, the resonances of these two cavities can be misaligned to further improve acoustic wave excitation. In addition, the coupling between the Si substrate cavity and the SiO_2_ and AlN cavities causes periodic fluctuations of the FSR and higher-order dispersion, as will be discussed later.

The AOM of the microring resonator can be characterized by an optomechanical S_21_ measurement as shown in Fig. 2g. By setting the input laser frequency (~ 1550 nm) at the slope of the optical resonance, the output laser intensity is modulated when a −5 dBm RF signal from port 1 of the vector network analyzer is launched on the AlN. The intensity modulation of the optical signal is measured using a photodiode and sent back to port 2 for the S_21_ measurement. It is noteworthy that no optical and electrical amplifiers are employed to preserve direct electro-opto-mechanical transduction. The optomechanical S_21_ measurements are performed for both the transverse electric (TE) and transverse magnetic (TM) optical modes, as shown in Fig. 2b, c, respectively. As expected, periodic peaks are observed, which correspond to each HBAR mode in S_11_. Due to the different optical mode profiles of the TE and TM modes (and thus acousto-optic mode overlaps) and optical *Q* factors, they respond differently, with the strongest response around 2 GHz for the TE mode and around 4 GHz for the TM mode. As the TE mode shows higher optical *Q*, it can enter the resolved sideband regime at frequencies beyond its optical linewidth (1–2 GHz), where the modulation sidebands are suppressed when biasing at the resonance slope. The zoom-in of highlighted regions (green shaded areas) are as shown in Fig. 2e, f, illustrating clear peaks with high contrast (>20 dB) between resonance and anti-resonance. Interestingly, the same measurements are performed for devices with a different actuator lateral shape, which show similar results for S_11_ and S_21_ of the TE and TM modes (see Supplementary Note 2). This indicates that the HBAR mode distribution and optomechanical spectra are less related to the shape of the actuator, but more to the vertical stack. This relaxes the requirements on actuator shape and size, which lends itself with high design freedom and small footprint.

A numerical Finite Element Method (FEM) simulation of one typical acoustic mode at 2.041 GHz is shown in Fig. 2h and a zoom-in around the optical waveguide is in Fig. 2i. The acoustic standing wave distributes uniformly through the entire substrate, which indicates that the optical circuits can be buried deeply inside the SiO_2_ cladding, free from the trade-off between actuation efficiency and absorption losses due to metal as found in traditional optical modulators. Also, due to the orders of magnitude smaller velocity of acoustic waves, the acoustic wavelength at microwave frequencies is comparable to the optical wavelength and waveguide structure. Interestingly, this is achieved with macroscopic actuators, as the acoustic wavelength is primarily dictated by the material stack and thickness, rather than lithography. Evidently, the simplicity of our structure presents advantages such as simple fabrication, high fabrication tolerance, high rigidity, and high power handling. In addition, it can be seen from Fig. 2h that the acoustic mode is largely confined beneath the actuator which guarantees low electromechanical cross-talk (−60 dB) between adjacent modulators (see Supplementary Note 3). This helps us to place multiple actuators on the same optical ring for compact spatiotemporal modulation as will be discussed in the last section. All these features may supplement the conventional SAW-based AOM for future microwave photonics applications where ultra-high optical Q, low cost, and dense integration are required.

### Mechanical mode dispersion

An analytical electromechanical model is established to help us get deep understanding of the mechanical performance of the device (see Supplementary Note 1). As mentioned above, the coupling from SiO_2_ and AlN cavities not only modulates the resonances’ magnitude envelope but also the dispersion of mechanical modes (deviation from equidistant spectrum). Figure 2j clearly shows the variation of frequency difference (local FSR) between each pair of adjacent resonances. The local FSR varies nearly periodically around an average value of 17.5 MHz with the same period as the envelope in Fig. 2a. By comparing Fig. 2a, j, it can be found that at each node of the envelope, the FSR reaches maximum value, which means the spacing of Si resonances increases near the SiO_2_ resonance. From the standpoint of the Si cavity, the variation of FSR can be understood intuitively from the fact that, the wave reflected back into the Si substrate from the Si-SiO_2_ interface experiences a varying phase shift at different frequencies. For example, around the SiO_2_ resonance, the Si-SiO_2_ interface locates at the node of an acoustic stress wave with near-zero stress (maximum displacement), which presents a free boundary condition. Far beyond the SiO_2_ resonances, the interface is at an anti-node corresponding to fixed boundary condition (zero displacement). These various boundary conditions each impose a different phase for a given reflected wave and thus change the effective cavity length of Si.

However, for the AlN and SiO_2_ cavities, the green dashed lines that denote the location of each SiO_2_ resonance lie closely together around 4 GHz where the AlN cavity resonance is located. Also, it can be seen that the average FSR of the acoustic modes decreases around the AlN resonance. Based on these observations and the fact that the acoustic impedance of SiO_2_ is smaller than Si and then AlN, we can conclude that, for two coupled acoustic cavities, the small cavity (e.g., SiO_2_) with smaller acoustic impedance tends to decrease (increase) the effective cavity length (FSR) of the big cavity (e.g., Si) when it’s on resonance compared with off-resonance and vice versa^38^. This is similar to coupled optical cavities by treating acoustic impedance as the effective refractive index^39^.

The higher order dispersion is presented in Fig. 2k, which shows the frequency deviation of each resonance from the ideally even distribution grid with reference to mode at 3.0145 GHz and period of 17.515 MHz. Mathematically, it can be interpreted as the integral of Fig. 2j (i.e., the accumulation of FSR deviation relative to 17.515 MHz) with respect to the origin at 3.0145 GHz. The higher order dispersion also shows periodic variation caused by the coupling between the Si and SiO_2_ cavities, which varies between normal and anomalous group velocity dispersion. The roll-off starting around 3.5 GHz is caused by reduced FSR due to coupling of the AlN cavity. This study of mechanical dispersion could benefit the growing field of mechanical dispersion engineering for future applications (e.g., mechanical dissipative solitons^40,41^, HBAR-based quantum random access memory^39,42^) and future devices improvement by optimizing Si, SiO_2_, and AlN thicknesses or by choosing materials with different acoustic impedance. In this sense, further theoretical and numerical studies are necessary for a more complete understanding of the acoustic wave propagation and mechanical cavity coupling in such a platform.

### Ultra-fast stress-optical actuation

**Fig. 3:**
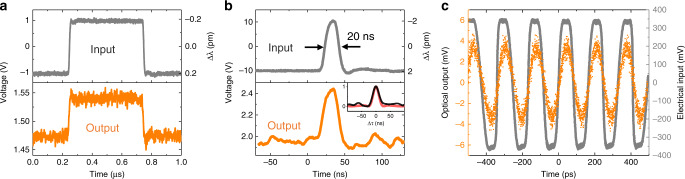
High-speed temporal modulation. **a** Time domain response (orange) to a small-signal square wave (gray) with 2 V *V*_pp_, 1 MHz repetition rate, and a 50% duty cycle. The sharp peaks at tuning edges are caused by mechanical ringing of acoustic resonances. **b** Time domain response to short pulses with 20 V *V*_pp_, 5 MHz repetition rate, and a 20 ns pulse width, demonstrating ultra-fast (sub-ns) tuning speed. The inset shows the normalized cross-correlation (black) between input and output signals and the auto-correlation (red) of the input signal. The right *Y* axis in **a**, **b** denotes the resonant wavelength shifting relative to 0 V voltage, according to a measured linear tuning of −0.2 pm V^−1^. **c** Response to a 6 GHz square wave driving at the frequency where mechanical resonances disappear due to the low mechanical *Q*. The electrical input signal (gray) is measured by an oscilloscope after 20 dB attenuation of the original signal. The optical output (orange) shows clear oscillations with a frequency equal to the driving field, illustrating GHz level piezoelectric actuation.

Besides the resonant microwave frequency AOM, the proposed stress-optical platform is capable of ultra-fast tuning of optical resonances with ultra-low power consumption. Here, the actuation speed is fully explored, showing sub-ns response time. To do this, the time domain dynamic response is recorded while applying a modulated signal. A small-signal (*V*_pp_ = 2 V) square wave (1 MHz) is first applied in Fig. 3a and the transmitted light intensity modulation is measured. Although with only 0.4 pm relative resonant wavelength shifting, the output signal shows clear separation between the two switching states, which is larger than the noise level. This makes it possible for feedback control of Kerr microcombs based on small error signals. We also observe sharp peaks at tuning edges when rapidly switching from one state to the other, which is caused by the excitation of acoustic resonances. These resonances can be damped by roughing the backside surface of the substrate for modulation with wider bandwidth (see Supplementary Note 4).

Periodic (5 MHz) short pulses with 20 ns pulse width are also applied as seen in Fig. 3b. The response follows in good shape with the rising and falling edges with the input pulse. To quantitatively show the similarity between input and output signals, the normalized cross-correlation between them is calculated as shown in the inset of 3b. Indeed, the similarity between the auto-correlation (red) of the input signal itself and the cross-correlation (black) demonstrates ultra-fast actuation beyond the nanosecond.

Despite the high actuation speed, the existence of HBAR modes prevents digital modulation at repetition rates beyond 1 GHz, where a flat and broadband response is usually required as in traditional optical communication. In this sense, pre-conditioning of the input signal or data post-processing is necessary to eliminate the distortion from mechanical resonances. However, due to the constant *f* ⋅ *Q* product in a general mechanical resonant systems, the acoustic resonances at high frequency gradually disappear due to the low mechanical *Q*. If we apply an ultra-high repetition rate (*f*_rep_) signal in the mechanical resonance damping-out region, we can get rid of the mechanical resonance induced signal distortion, in an attempt to find the ultimate speed limit of the actuator itself. This is demonstrated in Fig. 3c, where a 6 GHz square wave (gray curve) is applied using a programmable pattern generator and amplified by an optical modulator driver to *V*_pp_ of 7 V (21 dBm in power). The output optical modulation (orange) shows distinguishable oscillation at the same repetition rate, which suggests that the piezoelectric actuator itself can perform an ultra-fast actuation, despite the fact that broadband modulation is mostly limited by acoustic resonances. Because of the lower responsivity to higher harmonic Fourier components (e.g., 12 GHz, 24 GHz) of the input square wave, the output behaves more like a 6 GHz sinusoidal wave. It is noteworthy that the measured 8 mV *V*_pp_ corresponds to −38 dBm in power, which suggests an efficiency of −59 dB from RF modulator input to RF output at the photodetector. This modulation efficiency is still far away from the state of the art of electro-optical modulation^43^. Further measures are currently underway to improve the efficiency such as shrinking the device size and releasing the oxide membrane for better confinement of acoustic energy.

### Silicon Nitride optical isolator

The demonstrated time modulation, together with tight spacial localization, enables compact spatiotemporal modulation for studying topological photonics. As a demonstration, we realized, for the first time, an optical isolator built on Si_3_N_4_ integrated photonics using angular momentum biasing^44^. Optical nonreciprocal devices, such as circulators and isolators, are key building blocks in applications such as signal routing in telecommunication, reflection rejection in high-power lasers, and noise rejection in optical interfaces with superconducting qubits. Conventionally, these blocks are realized using the Faraday effect of magneto-optical materials under magnetic field^45^, which are challenging to integrate on photonic integrated circuits (PICs) and not compatible with superconducting circuits.

Recently, several magnet-free methods have been employed to demonstrate nonreciprocity on chip by breaking the time-reversal symmetry using optical^46^ or optomechanical^47^ nonlinearities, and stimulated Brillouin scattering^48,49^, which rely on additional high-power pump laser. It has been theoretically proposed to achieve optical nonreciprocity by momentum biasing through spatiotemporal modulation^15^, which can be purely electrically driven and independent of the input laser power. Experimentally, this concept has been demonstrated with integrated AlN photonics using SAW modulation^50^ for linear momentum biasing. However, the isolation is limited by the maximum applicable RF power to the fragile sub-micron electrode fingers. Here, using our HBAR platform we realize angular momentum biasing instead^44^, by placing multiple modulators within a single optical microring resonator to create a circulating refractive index modulation. This scheme benefits from significantly higher power handling capabilities of AlN actuators and thereby improves the attainable isolation.

**Fig. 4:**
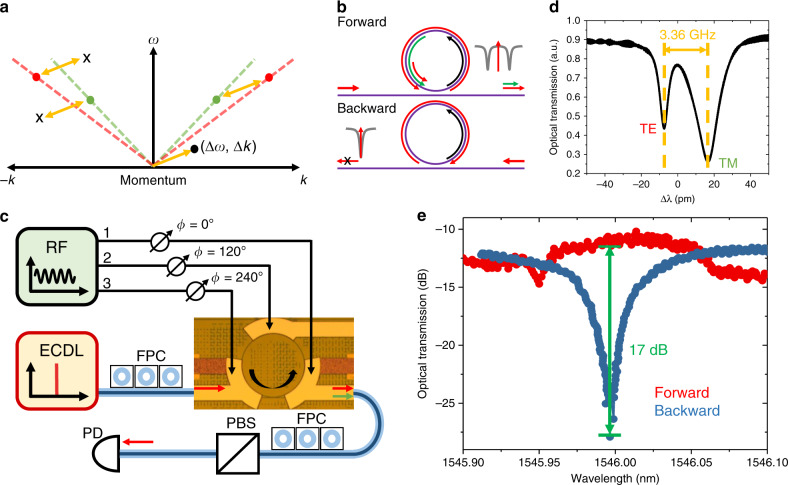
Si_3_N_4_ optical isolator via spatiotemporal modulation. **a** Schematic showing directionally dependent coupling between two optical modes *a* and *b* in *ω*–*k* space, induced by a rotating modulation wave. **b** In the forward direction, light transmits at resonance due to mode splitting from the strong coupling between modes *a* and *b*. Light in the backwards direction, however, will be absorbed due to phase mismatch and inefficient mode coupling. **c** Simplified schematics of measurement setup. The inset is the optical microscopy image of the fabricated device. Three AlN piezoelectric actuators are driven with fixed phase differences, generating a synthetic circulating wave along the ring. FPCs control the polarization of input and output light, and the polarization beam splitter (PBS) filters out unwanted modulation sidebands. **d** Optical transmission showing one pair of TE and TM resonant modes that are closely spaced by only 3.36 GHz. **e** Optical transmission of TE mode in forward and backward directions (with respect to off-chip input laser of −3 dBm) under +36 dBm RF driving. A 17 dB isolation is achieved at resonance with a bandwidth of 1 GHz.

The nonreciprocity in our device is induced by the direction dependent coupling between two optical modes (*a* and *b*) of a ring by the dynamic modulation^15^. To couple the two optical modes efficiently, both energy and momentum conservation need to be satisfied as shown in the *ω*-*k* space in Fig. 4a. This can be achieved by driving a rotational modulation wave along the ring whose frequency equals the frequency difference of the two optical modes. Meanwhile, the modulation wave carries a positive momentum *Δk* in the counter-clockwise (CCW) direction. Thus, the light in the ring can only be scattered in *ω*-*k* space along the vector that is predefined by the spatiotemporal modulation. Taking mode *a* for example, in the forward direction (positive *k*), light injected in mode *a* will be scattered into mode *b*, which can also be scattered back. For sufficiently strong driving, where the rate of energy exchange between the two optical modes exceeds their loss rates, there will be Rabi mode splitting and transparency at resonance^15^ (Fig. 4b). This can be understood qualitatively as the result of interference of the light that is directly coupled from the bus waveguide and the one that is first converted to the mode *b* and then converted back. At resonance, the phase difference between the two optical paths is *π* and thus destructively interfere. For the light in the bus, it will transmit without seeing the ring. On the other hand, if the light is in the backward direction and thus clockwise (CW) (negative *k*) in the ring, the scattering between the two modes is forbidden due to momentum mismatch. The ring will then show the original resonance dip and absorb all the light at critical coupling. Similar results can be obtained when we probe mode *b*.

Figure 4c shows the image of the fabricated device. Fundamental TE and TM polarization modes are chosen, as they can be easily engineered to have close frequencies and controlled experimentally using polarization controller. The spacing between a pair of TE and TM modes is found by measuring the optical transmission as inputting a mixture of TE and TM polarization light (Fig. 4d), where 3.36 GHz is obtained. One of the HBAR modes around the spacing frequency is excited to temporally modulate the ring. The clear separation ensures to work at resolved sideband regime where the modulation frequency is larger than the optical linewidth. Also, since the optical FSR is 200 GHz and the waveguide is designed to only support fundamental TE and TM mode families, the other undesired modes are far away from the two closely spaced TE and TM modes. Therefore, most energy scattering will be between the two modes of interest, and the probability of scattering to undesirable modes is low. The spatially varying modulation, required for momentum conservation, is realized by placing three discrete actuators that are equally spaced along the ring. By driving them with fixed phase differences (0°, 120°, 240°), an effective rotational wave is formed circulating around the ring, which induces coupling between the two optical modes only in CCW direction. More details about the effect of discrete modulation regions and sensitivity to RF relative phases can be found in the Supplementary Note 5.

In measuring the nonreciprocity, the polarization of input light is aligned to couple to TE mode only, and the output converted TM light is filtered with only TE light measured. Under +36 dBm RF driving power on each actuator, 17 dB isolation contrast at resonance between two directions is achieved as shown in Fig. 4e. The isolation bandwidth of 1 GHz is mainly limited by the linewidth of the optical TE resonance. The maximum transmission in forward direction is around −10 dB. It is noteworthy that this is the off-chip insertion loss, which includes −4 dB loss per fiber to chip coupling facet, and fiber and connectors losses. As the on-chip waveguide loss is −0.1 dB mm^−1^, the on-chip insertion loss is around −0.5 dB under 5 mm chip length. The high RF power can be further reduced in the future by increasing the optical *Q* using optimized photonic Damascene process^51^ (~8 dBm is enough for 5 million *Q*, corresponding to 1.6 V *V*_pp_, which is CMOS compatible). By cascading the device into multiple coupled rings, our platform enables to investigate experimentally new physical phenomena, such as synthetic dimensions^16^ and Floquet dynamics in topological photonics^17^, which rely on modulation in both time and space domain.

## Discussion

In conclusion, we demonstrate the integration of HBAR resonators within a nanophotonic platform. We realize a hybrid multi-functional stress-optical platform based on bulk acoustic waves. In the device, acoustic resonances are effectively generated by a macroscopic AlN actuator, and are used for microwave frequency AOM. These acoustic modes are evenly distributed over a broad frequency range up to 6 GHz. As an application, we realize an optical isolator in Si_3_N_4_ PICs platform using spatiotemporal modulation of the ring resonator via localized HBAR modes. This could serve not only as an important building block in Si_3_N_4_ photonics, but may also open up new pathways to investigate novel topological photonics. The same platform could also be engineered for quantum microwave to optical conversion^39^, which together with the isolator could act as an optical interface for superconducting circuits with high quantum efficiency and low noise cross-talk between the optical and microwave domain. Altogether, these advantages serve to produce a device which is not only novel, but has the potential for widespread applications across a variety of fields.

## Methods

### Device fabrication

The Si_3_N_4_ waveguides are fabricated using a subtractive process^37^. The fabrication process of AlN piezoelectric actuators is described here in detail. 100 nm Mo and 1 μm polycrystalline AlN films are sputtered on SiO_2_ cladding through foundry services (OEM Group). The AlN disk is patterned by AZ1518 photoresist and dry-etched using Cl_2_ and BCl_3_ in a Panasonic E620 Etcher. The dry etching of the bottom electrode (Mo) is performed using Cl_2_ in the same Panasonic E620 Etcher. Finally, the top 100 nm of Al is evaporated by a PVD E-beam evaporator and patterned using a standard lift-off process. The whole wafer is diced into individual chips by deep reactive ion etching (DRIE) followed by mechanical polishing of the Si substrate. This enables smooth chip facets for efficient coupling of light from lensed fiber to inverse waveguide taper at the edge. This three-mask, photolithographic-only fabrication leads to a low cost and high fabrication tolerance. Mechanical *Q* can be further increased in the future by conducting Chemical Mechanical Polishing (CMP) instead of mechanical polishing, which will produce sub-nanometer surface roughness.

### Experimental setup

The electromechanical S_11_ reflection response is measured by port 1 of a network analyzer (Agilent E8364B), where the electrical signal is applied to the device through an RF GS probe (GGB 40A-GS-150). For the optomechanical S21 measurment, around 100 μW continuous wave light from a diode laser (Velocity Tunable Laser 6328) is edge-coupled to the device using a lensed fiber via inverse taper. A −5 dBm RF electrical signal is applied from port 1 of the network analyzer to drive the piezoelectric actuator and the light intensity modulation is detected by a 12 GHz photodiode (New Focus 1544), which is sent back to port 2 of the network analyzer.

In the high repetition rate time domain modulation measurement, a 6 GHz square wave is supplied by a programmable pattern generator (Tektronix PPG1251), which is amplified by an optical modulator driver (WJ communication SA1137-2) to 7 V *V*_pp_ before applying to the device. The intensity modulation is detected by the same photodiode (New Focus 1544) and then recorded by a broad bandwidth oscilloscope (Tektronix DSA8200).

## Supplementary information

Supplementary Information

Peer Review File

## Data Availability

All relevant data are available from the authors upon reasonable request.
