# Supplementary material for: Hybrid integrated photonics using bulk acoustic resonators
Source: Nat Commun. 2020 Jun 17;11:3073. doi: 10.1038/s41467-020-16812-6 (PMC7299988; doi:10.1038/s41467-020-16812-6)
Supplement: Supplementary file 1 — Supplementary Information [file 41467_2020_16812_MOESM1_ESM.pdf]

# Supplementary Information: Hybrid Integrated Photonics Using Bulk Acoustic Resonators

Hao Tian[1], Junqiu Liu[2], Bin Dong[1], J. Connor Skehan[2], Michael Zervas[2], Tobias J. Kippenberg[2,†], Sunil A. Bhave[1,†]

[1]*OxideMEMS Lab, Purdue University, 47907 West Lafayette, IN, USA*

[2]*Institute of Physics, Swiss Federal Institute of Technology Lausanne (EPFL), 1015 Lausanne, Switzerland*

[†] *E-mail: tobias.kippenberg@epfl.ch, bhave@purdue.edu*

## Supplementary Note 1: Analytic analysis of electro-opto-mechanical response

**Electromechanical model of HBAR mode.** To better explain the electromechanical S11 response and mechanical dispersion shown in the main text, a one-dimensional (1D) analytic electromechanical model is established by combining the well-known Mason model [1,2] and the transfer matrix method [3]. As shown in Fig. 1(a), the acoustic wave is assumed to propagate bidirectionally along the z axis due to acoustic reflection at interfaces, such that the mechanical displacement $u(z,t)$ can be expressed as [1]:

$$u(z,t) = A^{+}e^{-j(kz-\omega t)} + A^{-}e^{j(kz+\omega t)} \tag{1}$$

where, $\omega$ and $k = \omega/v_{ac}$ ($v_{ac}$ is acoustic velocity) are the frequency and acoustic wave number respectively, and $A^{+}$ and $A^{-}$ are amplitudes for the bidirectional propagating waves. The wave is then related to the velocities $v$ and forces $F$ (or stress $\sigma$) at the two surfaces at $z_1$ and $z_2$, working as boundary conditions [1]:

$$v_1 = \frac{\mathrm{d}u(z_1)}{\mathrm{d}t} = j\omega(A^{+}e^{-jkz_1} + A^{-}e^{jkz_1}) \tag{2}$$

$$v_2 = \frac{\mathrm{d}u(z_2)}{\mathrm{d}t} = j\omega(A^{+}e^{-jkz_2} + A^{-}e^{jkz_2}) \tag{3}$$

$$F_1 = Sc\frac{\mathrm{d}u(z_1)}{\mathrm{d}z} = -jSck(A^{+}e^{-jkz_1} - A^{-}e^{jkz_1}) \tag{4}$$

$$F_2 = Sc\frac{\mathrm{d}u(z_2)}{\mathrm{d}z} = -jSck(A^{+}e^{-jkz_2} - A^{-}e^{jkz_2}) \tag{5}$$

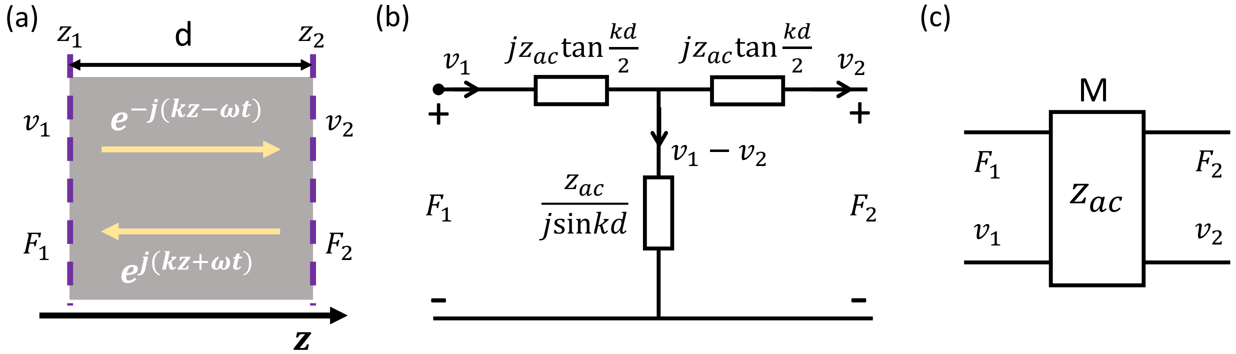


**Supplementary Figure 1:** Model of acoustic wave propagation in a non-piezoelectric material. (a) Acoustic wave propagates in forward and backward directions in a layer of non-piezoelectric material. The layer thickness is $d$ with two boundaries located at $z_1$ and $z_2$. The wave distribution is solely determined by the boundary conditions of force $F$ and velocity $v$. (b) Equivalent circuit model describing acoustic wave transmission. (c) The two boundaries can be correlated by a transfer matrix M which is a function of the acoustic impedance and the propagation length.

where $S$ is the surface area, and $c$ is the stiffness coefficient of the material . The prefactor related with time is omitted for simplicity. After some brief algebra, the forces can be expressed as the combination of velocities as:

$$F_1 = \frac{Z_{ac}}{j\text{sin}(kd)}(v_1 - v_2) + jZ_{ac}\text{tan}(\frac{kd}{2})v_1 \tag{6}$$

$$F_2 = \frac{Z_{ac}}{j\text{sin}(kd)}(v_1 - v_2) - jZ_{ac}\text{tan}(\frac{kd}{2})v_2 \tag{7}$$

where $Z_{ac}$ (=$S\rho v_{ac}$, where $\rho$ is the material density) is the acoustic impedance of the material, and $d$ is the thickness. Interestingly, if we treat force and velocity as voltage and current, an equivalent circuit model can be built which satisfies Eq. (6-7) according to Kirchhoff's law, as shown in Fig. 1(b). The circuit consists of three resistors with impedance as labeled in Fig. 1(b). Since the force and velocity must be continuous at the boundary between two different layers, the circuit model makes it easy to cascade different layers by connecting their corresponding circuits.

From Eq. (6-7), two adjecent boundaries can be related using a transfer matrix M as [3]:

$$\begin{bmatrix} F_1 \\ v_1 \end{bmatrix} = \begin{bmatrix} \cos(kd) & jZ_{ac}\text{sin}(kd) \\ j\text{sin}(kd)/Z_{ac} & \cos(kd) \end{bmatrix} \begin{bmatrix} F_2 \\ v_2 \end{bmatrix} = M \begin{bmatrix} F_2 \\ v_2 \end{bmatrix} \tag{8}$$

In this way, each layer can be represented by its characteristic transfer matrix M [Fig. 1(c)], and the relation between any two boundaries can be connected by multiplying the transfer matrix of each layer in between. The boundary condition at each interface can thus be determined from the very end boundaries of the entire stack structure which, for a general mechanical structure, satisfy the free boundary condition where the force is zero ($F = 0$), or the fixed boundary condition where the velocity (or equivalently the displacement) is zero ($v = 0$). After knowing the boundary conditions, the acoustic wave distribution in each layer can be determined from Eq. (2-5) by solving for $A^+$ and $A^-$. This is known as transfer matrix method for solving one-dimensional propagation of acoustic waves in multiple layer structures, which is suitable for our vertical stack structure of HBAR mode.

After the derivation of acoustic wave propagation, we are now ready for the model of acoustic wave excitation through a piezoelectric actuator. As we apply voltage to the piezoelectric material, the electric field will generate stress inside the film, which in turn builds up extra charges at the surfaces and change the electric field accordingly. The interplay between stress and electric field can be related as [1]:

$$\sigma = c^E \varepsilon + eE \tag{9}$$

$$D = e\varepsilon + \epsilon E \tag{10}$$

where $c^E$ is the stiffness coefficient under constant E, $e$ is piezoelectric coefficient, $\epsilon$ is dielectric constant, $\varepsilon$ is strain, E is electric field, and D is electric displacement. In general, the coefficients are matrices which correlate the mechanical and electric field in different directions. In our case, we consider only the terms related to the z direction.

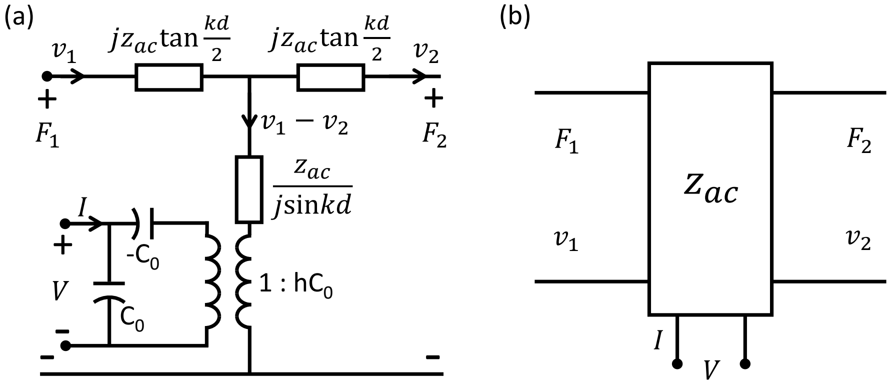


**Supplementary Figure 2:** Model of acoustic wave propagation in a piezoelectric material. (a) Equivalent circuit Mason model that describes the excitation and propagation of acoustic waves in the piezoelectric layer. The three resistors represent the propagation of acoustic waves, while the transformer represents energy conversion between electrical and mechanical domain. (c) Three ports representation of the piezoelectric actuator.

By performing a similar procedure as before, the velocities and forces generated at the boundaries can be calculated from the applied external voltage and current through [1]:

$$F_1 = \frac{Z_{ac}}{j\text{sin}(kd)}(v_1 - v_2) + jZ_{ac}\text{tan}(\frac{kd}{2})v_1 + \frac{h}{j\omega}I \tag{11}$$

$$F_2 = \frac{Z_{ac}}{j\text{sin}(kd)}(v_1 - v_2) - jZ_{ac}\text{tan}(\frac{kd}{2})v_2 + \frac{h}{j\omega}I \tag{12}$$

$$V = \frac{1}{j\omega C_0}[I + hC_0(v_1 - v_2)] \tag{13}$$

where $C_0$ (= $\epsilon S/d$) is the intrinsic capacitance of the piezoelectric actuator, and $h = e/\epsilon$ is a constant related with the material properties. Based on these equations, an equivalent circuit model can be established as shown in Fig. 2(a), which is the so-called Mason model [1]. Compared with the previous circuit in Fig. 1(b) which does not consider the piezoelectric effect, a transformer is added to the middle branch with a ratio of $1 : hC_0$, which then connects to the external power source through series and parallel capacitances $C_0$. The series capacitance has a negative sign which indicates that its current will combine with the external current $I$ and go through the parallel capacitance. This is to be consistent with Eq. (13). The other resistors describe the acoustic wave propagation in the piezoelectric layer as before. The piezoelectric layer can be treated as a three port component as shown in Fig. 2(b), where the mechanical ports are dependent on $I$-$V$ port. In transfer matrix method, this active component introduces additional boundary conditions through the electric port.

As we have the circuit model and transfer matrix of each layer, the actual device as described in the main text and shown in Fig. 3(a) can be modeled by simply connecting each adjacent layer.

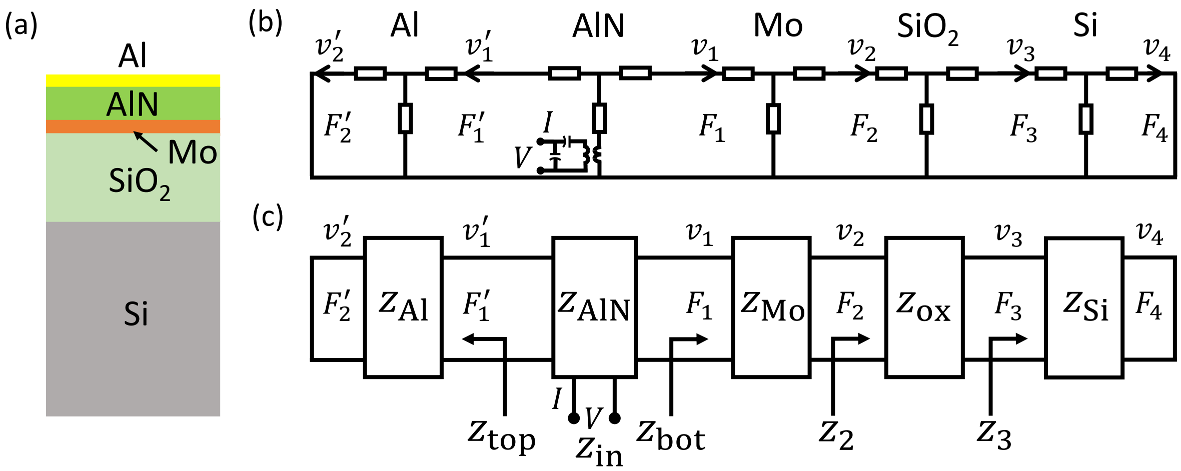


**Supplementary Figure 3:** Electromechanical model of the actual device in this work. (a) Vertical stacking structure of the whole device. (b) Equivalent circuit model by connecting adjacent layers. The end ports are shorted as required by the free boundary condition. (c) Transfer matrix chain that connects each interface. The input impedance at each port can be correlated and calculated by the multiplication of matrices in between.

Fig. 3(b) shows the equivalent circuit of the whole device. Free boundary conditions at the top and bottom surfaces are employed such that the forces $F'_2$ and $F_4$ are zero, which correspond to an electric short in the circuit. The impedance looking into one interface can be defined as $Z = F/v$. By utilizing Eq. (8), and assuming $F'_2 = 0$ and $F_4 = 0$, the impedance at port $1'$ and 3 from the top Al electrode and bottom Si substrate can be calculated easily as:

$$Z_{\text{top}} = jZ_{\text{Al}}\tan(k_{\text{Al}}d_{\text{Al}}) \tag{14}$$

$$Z_3 = jZ_{\text{Si}}\tan(k_{\text{Si}}d_{\text{Si}}) \tag{15}$$

where $Z_{\text{Al}}$ and $Z_{\text{Si}}$ are the acoustic impedance of Al and Si respectively. Similarly, by multiplying matrices of cascaded layers, the impedance at ports 2 and 1 can be calculated:

$$Z_2 = j\frac{Z_{\text{Si}}\tan(k_{\text{Si}}d_{\text{Si}}) + Z_{\text{ox}}\tan(k_{\text{ox}}d_{\text{ox}})}{1 - (Z_{\text{Si}}/Z_{\text{ox}})\tan(k_{\text{Si}}d_{\text{Si}})\tan(k_{\text{ox}}d_{\text{ox}})} \tag{16}$$

$$Z_{\text{bot}} = \frac{Z_2 + jZ_{\text{Mo}}\tan(k_{\text{Mo}}d_{\text{Mo}})}{1 + j(Z_2/Z_{\text{Mo}})\tan(k_{\text{Mo}}d_{\text{Mo}})} \tag{17}$$

where $Z_{\text{ox}}$ and $Z_{\text{Mo}}$ are the acoustic impedance of $SiO_2$ and Mo respectively. From Eq. (11-13), the electrical impedance $Z_{\text{in}}$ ($= V/I$) can be obtained by [1,2]:

$$Z_{\text{in}} = \frac{1}{j\omega C_0}\left\{1 - \frac{k_t^2}{k_{\text{AlN}}d_{\text{AlN}}}\frac{(z_{\text{top}} + z_{\text{bot}})\sin(k_{\text{AlN}}d_{\text{AlN}}) + j2[1 - \cos(k_{\text{AlN}}d_{\text{AlN}})]}{(z_{\text{top}} + z_{\text{bot}})\cos(k_{\text{AlN}}d_{\text{AlN}}) + j(1 + z_{\text{top}}z_{\text{bot}})\sin(k_{\text{AlN}}d_{\text{AlN}})}\right\} \tag{18}$$

where, $k_t^2$ is the intrinsic electromechanical coupling coefficient of AlN which is 6.5%, $z_{\text{top}}$ ($= Z_{\text{top}}/Z_{\text{AlN}}$) and $z_{\text{bot}}$ ($= Z_{\text{bot}}/Z_{\text{AlN}}$) are the impedance from the top and bottom side of AlN which are normalized by the AlN acoustic impedance $Z_{\text{AlN}}$.

**Electromechanical $S_{11}$ reflection parameter.** By applying actual material properties as summarized in Table 1, the electrical input impedance can be calculated, from which the $S_{11}$ response can be calculated as:

$$S_{11} = \frac{Z_0 - Z_{\text{in}}}{Z_0 + Z_{\text{in}}} \tag{19}$$

where $Z_0$ (50 Ω) is the standard normalized impedance of the network analyzer. The results are shown in Fig. 4 which demonstrates similarity between measurements and calculations from the electromechanical model. The difference in magnitude is mainly introduced by the calibration and parasitic capacitance from the probe landing during the electrical experiments. The variation of envelope caused by the coupling between Si, $SiO_2$ and AlN cavities is well captured by the model. As mentioned in the main text, the node of the envelope corresponds to a $SiO_2$ resonance. This is because, at the $SiO_2$ resonance, more acoustic energy is confined in $SiO_2$ which is softer and has smaller acoustic impedance as compared to Si.

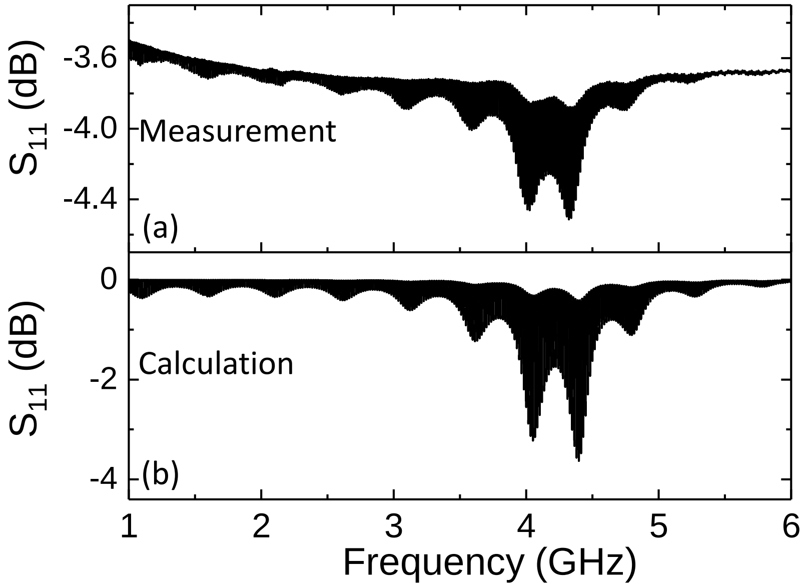


**Supplementary Figure 4:** Measured (a) and calculated (b) $S_{11}$ reflection parameter, showing much similarity in terms of varied envelope and its period.

**Mechanical dispersion analysis.** Provided the precision of the model demonstrated in last section, we can rely on the model and analyze the mechanical dispersion by calculating resonant frequencies. By letting the denominator in Eq. (18) equal zero, we retrieve the parallel resonant frequencies corresponding to maximum resistance [2]:

$$(z_{\text{top}} + z_{\text{bot}})\cos(k_{\text{AlN}}d_{\text{AlN}}) + j(1 + z_{\text{top}}z_{\text{bot}})\sin(k_{\text{AlN}}d_{\text{AlN}}) = 0 \tag{20}$$

The dispersion equation is transcendental and can be solved numerically. The FSR variation and higher order dispersion can thus be calculated from resonant frequencies as shown in the main text. To get a feeling of why and how the FSR varies, an analytic expression of FSR can be derived

under simplified assumptions. Specifically, we can assume the metal thickness is much smaller than the acoustic wavelength, such that their impedance is nearly zero. Under this assumption, the dispersion equation Eq. (20) can be simplified to:

$$Z_{\text{Si}}\tan(k_{\text{Si}}d_{\text{Si}}) + \frac{Z_{\text{ox}}\tan(k_{\text{ox}}d_{\text{ox}}) + Z_{\text{AlN}}\tan(k_{\text{AlN}}d_{\text{AlN}})}{1 - \frac{Z_{\text{AlN}}}{Z_{\text{ox}}}\tan(k_{\text{AlN}}d_{\text{AlN}})\tan(k_{\text{ox}}d_{\text{ox}})} = 0 \tag{21}$$

The equation is arranged in the way that $SiO_2$ and AlN are combined and work together as an external cavity coupled to the Si cavity. The FSR can be divided into four regions in terms of resonance and anti-resonance of $SiO_2$ and AlN cavities (see Table 2 and Fig. 5).

Firstly, let's consider frequencies around AlN resonance, where $k_{\text{AlN}}d_{\text{AlN}} = p\pi + \delta_{\text{AlN}}$, with $p$ an integer number and $\delta_{\text{AlN}}$ denoting a small deviation. When the $SiO_2$ is at resonance such that the second term in Eq. (21) is near zero, $k_{\text{ox}}d_{\text{ox}} = n\pi + \delta_{\text{ox}}$. In this case, Si cavity must satisfy $k_{\text{Si}}d_{\text{Si}} = m\pi + \delta_{\text{Si}}$, such that Eq. (21) becomes [2]:

$$Z_{\text{Si}}\delta_{\text{Si}} + Z_{\text{ox}}\delta_{\text{ox}} + Z_{\text{AlN}}\delta_{\text{AlN}} = 0 \tag{22}$$

From Eq. (22) we derive the relation between the three cavities as:

$$k_{\text{Si}}d_{\text{Si}} = m\pi - \frac{Z_{\text{ox}}}{Z_{\text{Si}}}(k_{\text{ox}}d_{\text{ox}} - n\pi) - \frac{Z_{\text{AlN}}}{Z_{\text{Si}}}(k_{\text{AlN}}d_{\text{AlN}} - p\pi) \tag{23}$$

where $k_{\text{Si}} = 2\pi f_m/v_{\text{Si}}$, $k_{\text{ox}} = 2\pi f_m/v_{\text{ox}}$, $k_{\text{AlN}} = 2\pi f_m/v_{\text{AlN}}$. This is also true for the $m-1$ mode. Note that the mode order $p$ and $n$ of AlN and $SiO_2$ will not change for the $m$ and $m-1$ modes. By taking the frequency difference between the $m$ and $m-1$ modes, we can get the local FSR after some algebra as:

$$\Delta f = \Delta f_0 \frac{d_{\text{Si}}}{d_{\text{Si}} + \frac{\rho_{\text{ox}}}{\rho_{\text{Si}}}d_{\text{ox}} + \frac{\rho_{\text{AlN}}}{\rho_{\text{Si}}}d_{\text{AlN}}} \tag{24}$$

where $\Delta f_0 = v_{\text{Si}}/(2d_{\text{Si}})$ is the original FSR of the Si cavity. This new FSR is smaller than the original FSR since the $SiO_2$ and AlN extends the effective cavity length. It is interesting to note that this extension is not simply the physical length of each layer but the effective length weighted by its density relative to Si.

Following a similar procedure, the FSR at $SiO_2$'s anti-resonance and around AlN's resonance can be obtained. In this time, the three cavities satisfy:

$$k_{\text{AlN}}d_{\text{AlN}} = p\pi + \delta_{\text{AlN}} \tag{25}$$

$$k_{\text{ox}}d_{\text{ox}} = n\pi + \pi/2 + \delta_{\text{ox}} \tag{26}$$

$$k_{\text{Si}}d_{\text{Si}} = m\pi + \pi/2 + \delta_{\text{Si}} \tag{27}$$

By inserting them into Eq. (21), it becomes [2]:

$$-\frac{Z_{\text{Si}}}{\delta_{\text{Si}}} + \frac{-\frac{Z_{\text{ox}}}{\delta_{\text{ox}}} + Z_{\text{AlN}}\delta_{\text{AlN}}}{1 + \frac{Z_{\text{AlN}}\delta_{\text{AlN}}}{Z_{\text{ox}}\delta_{\text{ox}}}} = 0 \tag{28}$$

Since $Z_{\text{AlN}}\delta_{\text{AlN}}$ is a very small term, we can ignore it and get:

$$\delta_{\text{Si}} = -\frac{Z_{\text{Si}}}{Z_{\text{ox}}}\delta_{\text{ox}} - \frac{Z_{\text{Si}}Z_{\text{AlN}}}{Z_{\text{ox}}^2}\delta_{\text{AlN}} \tag{29}$$

We can then retrieve the relation between the three cavities as:

$$k_{\text{Si}}d_{\text{Si}} = m\pi + \pi/2 - \frac{Z_{\text{Si}}}{Z_{\text{ox}}}(k_{\text{ox}}d_{\text{ox}} - n\pi - \pi/2) - \frac{Z_{\text{Si}}Z_{\text{AlN}}}{Z_{\text{ox}}^2}(k_{\text{AlN}}d_{\text{AlN}} - p\pi) \tag{30}$$

By taking the frequency difference between $f_m$ and $f_{m-1}$, we can find the local FSR as:

$$\Delta f = \Delta f_0 \frac{d_{\text{Si}}}{d_{\text{Si}} + \frac{Z_{\text{Si}}^2}{Z_{\text{ox}}^2}\left(\frac{\rho_{\text{ox}}}{\rho_{\text{Si}}}d_{\text{ox}} + \frac{\rho_{\text{AlN}}}{\rho_{\text{Si}}}d_{\text{AlN}}\right)} \tag{31}$$

By comparing Eq. (24) and (31), it can be seen that the extra effective length due to $SiO_2$ and AlN is now multiplied by the square of the ratio between the acoustic impedance of Si and $SiO_2$. Since $SiO_2$ has a smaller impedance than Si, the effective length is longer than before, and the FSR is thus smaller .

The cases where the AlN is near its anti-resonance condition $k_{\text{AlN}}d_{\text{AlN}} = p\pi + \pi/2 + \delta_{\text{AlN}}$ can be calculated in a similar manner which are summarized in Table 2. Note the $SiO_2$ resonance is defined as when there is no Si, and now its resonant condition becomes $k_{\text{ox}}d_{\text{ox}} = n\pi + \pi/2 + \delta_{\text{ox}}$ due to the $\pi/2$ phase introduced from the AlN–$SiO_2$ interface. Similar as before, the length (FSR) is longer (smaller) around $SiO_2$'s anti-resonance as compared to its resonance. By comparing cases between AlN resonance and anti-resonance regions, there is an additional factor of the ratio square between the impedance of oxide and AlN around AlN anti-resonance. Since AlN has a higher impedance than $SiO_2$, the effective length (FSR) around AlN anti-resonance regions is shorter (larger) than the corresponding region around AlN resonance. Based on the effective length expressions, we can find that, for two coupled mechanical cavities, if the impedance of the small cavity is smaller (e.g., Si and $SiO_2$), the effective length (FSR) is shorter (larger) around the small cavity resonance than its anti-resonance. This is also true for the reverse case (e.g., $SiO_2$ and AlN), where the small cavity has larger impedance, and the effective length (FSR) is longer (smaller) around AlN resonance [2].

The boundary conditions at the interfaces in each case are also summarized in Table 2. The AlN–$SiO_2$ interface is free at the AlN resonance and fixed at anti-resonance. This is also true for the $SiO_2$–Si interface. The four cases are numbered as 1-4 from top to bottom in Table 2 and labeled and compared with experiments as shown in Fig. 5. Around the AlN resonance, the FSR oscillates between 1 and 2, while at AlN's anti-resonance it varies between 3 and 4, which is consistent with experiments. Finally, as a rule of thumb, since the envelope and FSR are both related with the matching of acoustic impedance, the node (anti-node) of the $S_{11}$ envelope corresponds to larger (smaller) local FSR.

**Supplementary Table 1:** Material properties of each layer employed in the analytic model

| Material | Density $\rho$ (kg/m$^3$) | Velocity $v$ (m/s) | Thickness $d$ ($\mu$m) |
|---|---|---|---|
| Al | 2700 | 6300 | 0.1 |
| AlN | 3300 | 11050 | 0.92 |
| Mo | 10200 | 6636 | 0.1 |
| $SiO_2$ | 2200 | 5640 | 5.44 |
| Si | 2329 | 8430 | 231.5 |

**Supplementary Table 2:** Effective cavity length at different resonant conditions. The effective boundary condition at each interface is compared.

| $k_{\text{AlN}}d_{\text{AlN}}$ | $k_{\text{ox}}d_{\text{ox}}$ | $k_{\text{Si}}d_{\text{Si}}$ | AlN–$SiO_2$ | $SiO_2$–Si | Effective length |
|---|---|---|---|---|---|
| $p\pi$ | $n\pi$ | $m\pi$ | Free | Free | $d_{\text{Si}} + \frac{\rho_{\text{ox}}}{\rho_{\text{Si}}} d_{\text{ox}} + \frac{\rho_{\text{AlN}}}{\rho_{\text{Si}}} d_{\text{AlN}}$ |
| $p\pi$ | $n\pi + \pi/2$ | $m\pi + \pi/2$ | Free | Fixed | $d_{\text{Si}} + \frac{Z_{\text{Si}}^2}{Z_{\text{ox}}^2} \left( \frac{\rho_{\text{ox}}}{\rho_{\text{Si}}} d_{\text{ox}} + \frac{\rho_{\text{AlN}}}{\rho_{\text{Si}}} d_{\text{AlN}} \right)$ |
| $p\pi + \pi/2$ | $n\pi + \pi/2$ | $m\pi$ | Fixed | Free | $d_{\text{Si}} + \frac{\rho_{\text{ox}}}{\rho_{\text{Si}}} d_{\text{ox}} + \frac{Z_{\text{ox}}^2}{Z_{\text{AlN}}^2} \frac{\rho_{\text{AlN}}}{\rho_{\text{Si}}} d_{\text{AlN}}$ |
| $p\pi + \pi/2$ | $n\pi$ | $m\pi + \pi/2$ | Fixed | Fixed | $d_{\text{Si}} + \frac{Z_{\text{Si}}^2}{Z_{\text{ox}}^2} \left( \frac{\rho_{\text{ox}}}{\rho_{\text{Si}}} d_{\text{ox}} + \frac{Z_{\text{ox}}^2}{Z_{\text{AlN}}^2} \frac{\rho_{\text{AlN}}}{\rho_{\text{Si}}} d_{\text{AlN}} \right)$ |

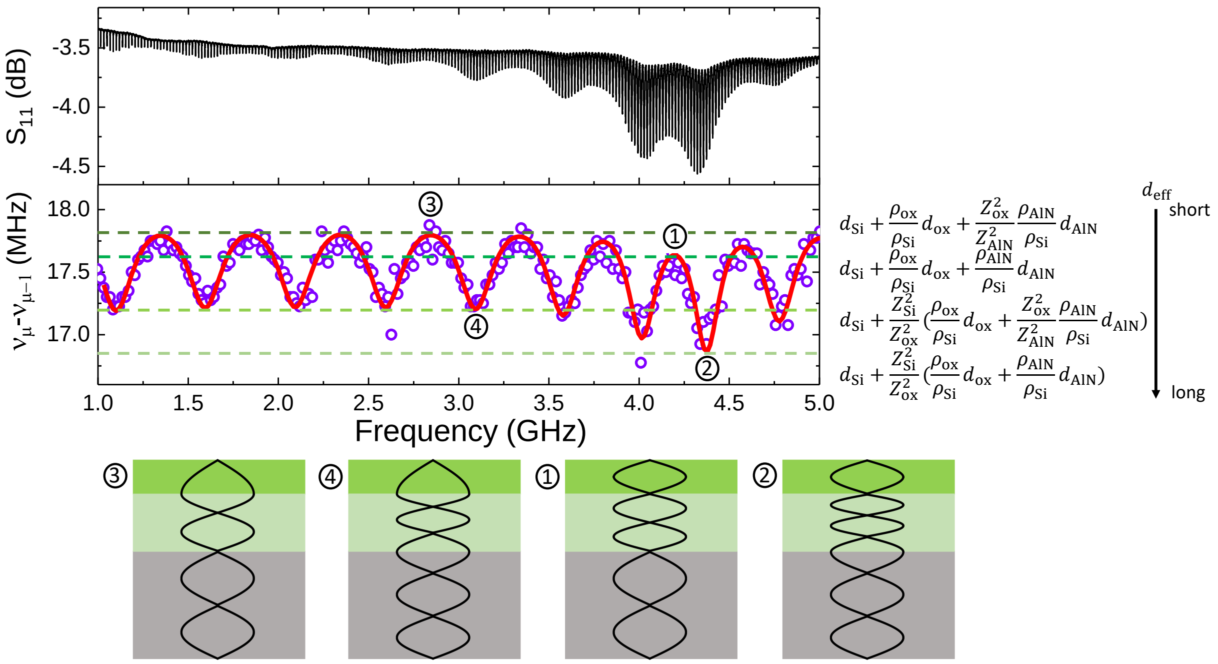


**Supplementary Figure 5:** Four different regions are labeled by number in the order from top to bottom corresponding to Table 2. The green dashed lines denote the FSR of each region and the corresponding effective length $d_{\text{eff}}$ is labeled on the right. The bottom insets show the schematics of the acoustic stress wave distribution for each region, illustrating the locations of interfaces relative to the stress wave.

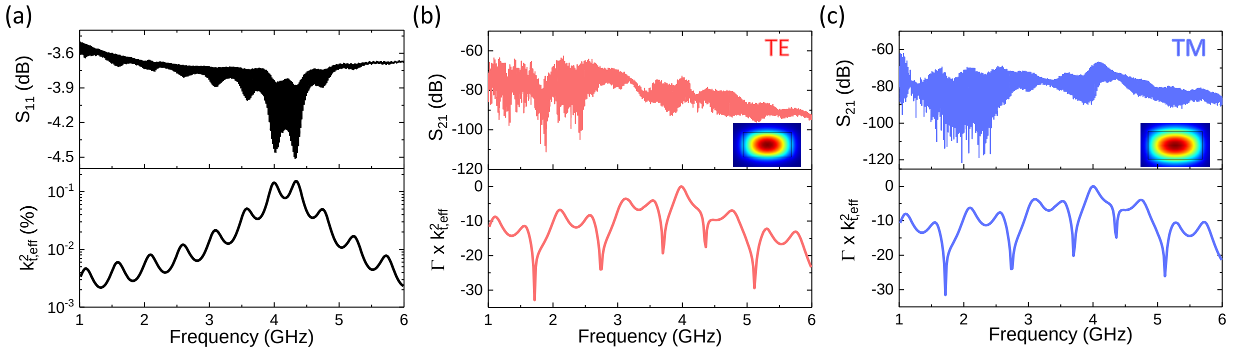


**Supplementary Figure 6:** (a) Measured $S_{11}$ response (top) and calculated effective electromechanical coupling coefficient $k^2_{t,eff}$ (bottom). The coupling reaches a maximum value of 0.15% around 4.3 GHz. (b-c) Measured $S_{21}$ response of the TE and TM mode (top) and corresponding normalized product of acousto-optic overlap and $k^2_{t,eff}$ (bottom). When the node of the acoustic stress wave is located at the center of the waveguide, the acousto-optic overlap integral $\Gamma$ becomes zero and thus causes notches in the $S_{21}$ response.

**Electromechanical coupling and acousto-optic overlap.** The model can also be used to estimate the optomechanical $S_{21}$ response which is dependent on both electromechanical coupling efficiency and acousto-optic overlap. The stress field distribution in AlN will strongly influence the effective electromechanical coupling, which can be estimated by following the method, as found in the literature [2]:

$$k^2_{t,eff} = \frac{\pi^2}{4}\frac{f_s}{f_p}(1 - \frac{f_s}{f_p}) \tag{32}$$

where $f_p$ is the parallel resonant frequency when the denominator of Eq. (18) equals zero, and $f_s$ is the series resonant frequency when the numerator of Eq. (18) equals zero. $k^2_{t,eff}$ is thus calculated as shown in Fig. 6(a). A maximum value of 0.15% is reached around 4.3 GHz where the AlN resonance is located. It varies with a envelope similar as the $S_{11}$ measurement.

In addition to elecromechanical conversion efficiency, the S21 also depends on acousto-optic overlap which determines the modulation of the optical resonant frequency. According to the perturbation theory, the relative change of resonant frequency can be related with the modulation of refractive index distribution as [4]:

$$\frac{\Delta\omega}{\omega} \approx -\frac{\iint \Delta n(x,y)|\mathbf{E}(x,y)|^2 \mathrm{d}x\mathrm{d}y}{\iint n(x,y)|\mathbf{E}(x,y)|^2 \mathrm{d}x\mathrm{d}y} \tag{33}$$

where the perturbation of refractive index is caused by the induced stress through the stress-optical effect, and is proportional to the stress via the stress-optical coefficient. In our specific case, the stress is dominated by the vertical stress $\sigma_z$. Therefore, the normalized acousto-optic overlap can be approximately estimated as [4]:

$$\Gamma = \frac{\iint \sigma_z(z)|E(r,z)|^2 \mathrm{d}r\mathrm{d}z}{\iint |E(r,z)|^2 \mathrm{d}r\mathrm{d}z} \tag{34}$$

where $\sigma_z$ is assumed to be dependent only on $z$ and uniform in the $r$ direction. In this way, the distribution of $\sigma_z$ can be calculated from the aforementioned 1-D acoustic model. The electric field takes only the dominant component, that is $E_r$ for TE mode and $E_z$ for TM mode.

To better compare with the measured optomechanical $S_{21}$ response, the normalized product of $\Gamma$ and $k_{t,eff}^2$ is plotted in Fig. 6 (b-c) for TE and TM modes. Their product can help us to explain the variation of $S_{21}$ qualitatively. For instance, when the node of a stress wave locates at the center of the waveguide, the overlap integral approaches zero due to the vertical symmetry of the optical mode. This causes notches in the $S_{21}$ response, such as the decreasing of $S_{21}$ near 2 GHz of the TE mode. The nearly periodic variation of the envelope of $S_{21}$ is due to the modulation of $k_{t,eff}^2$. Due to the high confinement of the optical mode in the waveguide, there exhibits only a small difference of the acousto-optic overlap between the TE and TM modes. On the other hand, the difference of the measured $S_{21}$ response is mainly caused by the optical Q. Since TE mode shows nearly two times larger Q than TM mode, its response beyond 4 GHz is suppressed by entering into the resolved sideband regime. The perturbation of the local stress field due to the $Si_2N_4$ waveguide is not taken into account which requires 2D numerical simulation. This may lead to the difference between measurement and calculation shown in Fig. 6(b-c). Despite this, the analytic model can still give us a good qualitative estimation of the electro-optomechanical response.

**Supplementary Note 2: Investigation of different actuator shapes**

Devices with different actuator shape are fully explored, and its influence on DC optical resonance tuning and high frequency modulation is investigated. The original design with a disk shaped actuator as described in the main text is shown in Fig. 7(a), which is refered as the Disk device in the following text. Since the stress mainly originates from the corner of the actuator, the microring resonator is positioned at the outer edge of the disk actuator. Under positive DC biasing on the top electrode (while the bottom electrode is always grounded), the AlN film expands and pushes the ring outwards (i.e., ring expanding) [Fig. 7(e)]. Intuitively, the other design is to use a donut-shape actuator which squeezes the microring resonator if it is placed at the inner edge of the actuator, as shown in Fig. 7(b). This is referred as the Donut device in the following. These two designs will show different stress distribution and static resonance tuning as demonstrated later. The cross-sections for the two designs are illustrated in Fig. 7(c-d). The ring is placed 3 µm within the edge of the Disk actuator, while 5 µm for the Donut actuator. The final fabricated devices are shown in Fig. 7(g-h) for the two designs. False color SEMs zoom-in around actuator corners in Fig. 7(i-j) show the relative position between the optical microring resonator (blue) and the AlN actuator (green).

The static mechanical simulation is conducted using the finite element method (COMSOL) as shown in Fig. 7(e-f), in which +60 V DC biasing is applied on the top Al layer while the bottom Mo layer is grounded. In this case, a negative electric field $E_z$ forms (points downwards) across the AlN thin film, and the AlN film will be squeezed in the z-direction and expand horizontally

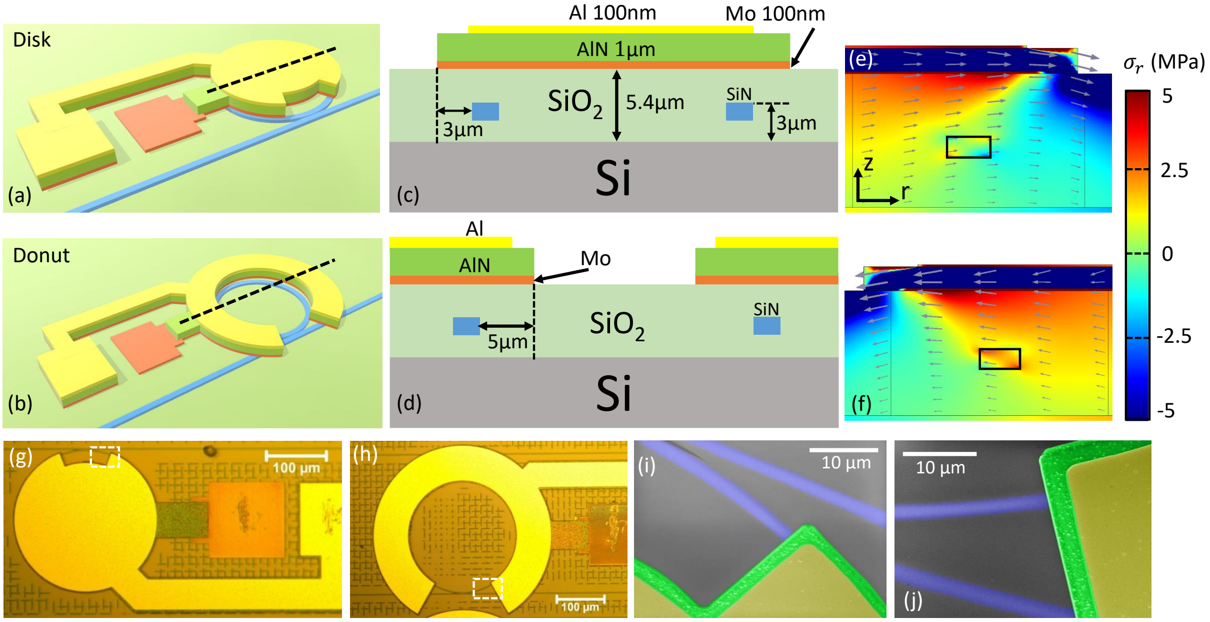


**Supplementary Figure 7:** Schematics for the designed devices with (a) Disk shape and (b) Donut shape actuators with the silicon nitride ring resonator (blue) having different relative positions. (c) and (d) are the cross-sections for the Disk and Donut devices along black dashed lines in (a) and (b), respectively. (e-f) COMSOL simulation of horizontal stress distribution around the optical waveguide under +60 V DC biasing for the Disk and Donut devices respectively. The overlaid gray arrows denote the local mechanical displacement with the biggest arrow scaled to 1 nm. (g-h) Optical microscope images of fabricated devices. (i-j) False color scanning electron micrograph (SEM) images around the actuator corner as labeled in white dashed box in (g) and (h) respectively with color similar to the cross-sections in (c-d).

(positive Poisson ratio). Fig. 7(e) illustrates the horizontal stress distribution and mechanical displacement around the optical waveguide at the upper right corner of Fig. 7(c). As AlN expands, starting from the corner, the stress splits into two parts: extensional stress under the actuator and compressing stress at outside, and the mechanical displacement orients mainly horizontally and points outside the actuator. Similar results can be drawn for Donut device as in Fig. 7(f). Depending on the position of the waveguide, it experiences extensional, compressing, or the interplay between these two. For the Disk device, the horizontal stress inside the waveguide is a mixture of extension and compression, while for the Donut device, it is mainly extension.

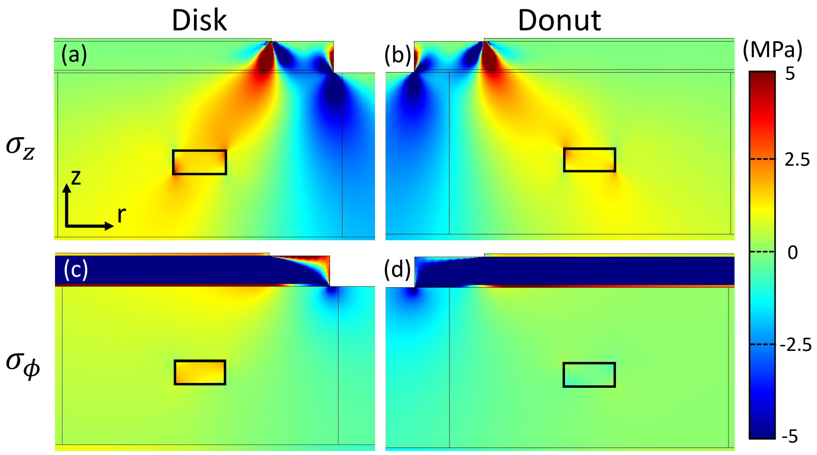


**Supplementary Figure 8:** Static stress distribution in z and $\phi$ directions. (a) and (b) show the numerical simulation of vertical (z) stress distribution under +60 V DC biasing for the Disk and Donut devices, respectively. Both of them show extensional stress around the waveguide. (c) and (d) are out of plane (tangential to optical ring) stress distributions for Disk and Donut devices. They present different sign inside the waveguide, since the optical ring of the Disk device expands whereas the Donut device squeezes.

Besides the dominant horizontal stress, stresses in other directions will also affect the modulation on the refractive index. As shown in Fig. 8(a) and (b), originating from the corner of top metal, $\sigma_z$ exhibit two main lobes with different sign. Inside the actuator, the waveguides in both cases experience extensional stress around 2 MPa. Under positive biasing, the ring of the Disk device will be pushed outwards as the actuator expands, which builds up extensional stress in the waveguide as in Fig. 8(c). On the other hand, at the inner edge of the Donut actuator, the optical ring is squeezed, generating compressing stress [Fig. 8(d)]. Since shear stress is found to play a less role compared with normal stress[5], it is not taken into account in this study. Additionally, when applying negative voltages, the AlN actuator changes from expanding (horizontally) to shrinking, so that all the stresses in the above analysis change sign. In this way, bi-directional tuning can be achieved by reversing the applied voltage's sign, as demonstrated in the following section.

The presence of stress will change the refractive index of optical material, and affect differently for distinct polarization of the electric field of light waves, causing so-called birefringence.

Numerically, they are correlated by stress-optical coefficients by [5]:

$$n_r = n_0 - C_1\sigma_r - C_2(\sigma_\phi + \sigma_z) \quad (35)$$
$$n_\phi = n_0 - C_1\sigma_\phi - C_2(\sigma_z + \sigma_r) \quad (36)$$
$$n_z = n_0 - C_1\sigma_z - C_2(\sigma_r + \sigma_\phi) \quad (37)$$

where, $n_0$ is original refractive index of the material, $C_1$ relates the refractive index and stress that are in the same direction while $C_2$ relates the two that are orthogonal. These equations are applicable to isotropic material such as amorphous $Si_3N_4$ from low-pressure chemical vapor deposition (LPCVD) used in this work. The lack of the stress-optical coefficient for $Si_3N_4$ in the literature makes it difficult to predict precisely the response of the optical ring resonator. However, it would be possible to extract the coefficients by comparing experimental tuning of optical ring resonator with simulation, which is under investigation.

**DC optical resonance tuning.** Working as a $Si_3N_4$ ring resonator tuner, the static optical resonance tuning is performed by applying DC biasing. Fig. 9(a) shows the transmission spectrum of one resonance under different voltages for both the TE and TM modes of the Disk device. One can see that as we apply a positive 60 V the resonance shifts to shorter wavelength (blue curve) relative to the original resonance (black curve), and the tuning changes direction after reversing the voltage (red curve), demonstrating bi-directional tuning ability. Also, it can be observed that the resonance dip only shifts horizontally with little changes of vertical depth, since the waveguide coupling region is unaffected by the opening section of the actuator. The dependence of resonant wavelength on voltages is summarized in Fig. 9(c), showing high linearity. Both TE and TM modes demonstrate similar tuning performances with nearly -12 pm under positive 60 V. This tuning range is on a similar order or larger than the linewidth of the high optical Q resonances, which is applicable for $Si_3N_4$ microcomb applications such as Kerr comb generation and stabilization.

More interestingly, due to different relative positions of ring resonator, the Donut device shows opposite behaviour: the resonant wavelength increases for positive voltages and vice versa, as can be seen in Fig. 9(b) and (d). Here, the slope of tuning with respect to voltage changes from negative to positive. On the other hand, it shows much smaller tuning range with 5.66 pm for the TM mode and 1.63 pm for the TE mode under +60 V. The different tuning range of two orthogonal polarization modes, TE and TM, demonstrates tunable birefringence in an otherwise isotropic material by controlling stress, which can be utilized for polarization control [6] or tuning the mode spacing and coupling between a pair of TE and TM modes in a ring resonator. The effect of mechanical actuation on optical Q is plotted in Fig. 9(e-f), which is found to be insignificant. The TE mode shows much higher Q than the TM mode, since the TM mode extends further in the vertical direction which is prone to the absorption of the bottom metal.

**RF frequency modulation of the Donut-shape device.** The same measurements described in the main text are also done for the Donut device, including $S_{11}$ and $S_{21}$ responses as shown in Fig. 10. No big differences can be found between the Disk and Donut devices in terms of mode

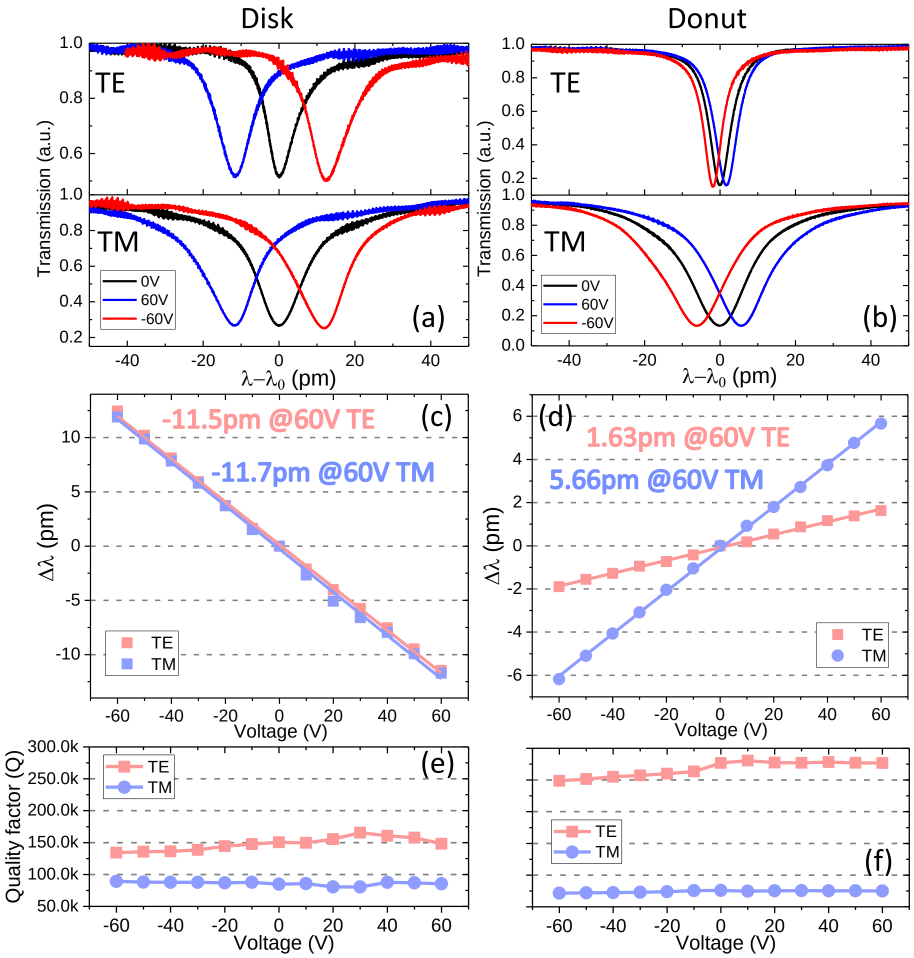


**Supplementary Figure 9:** Transmission spectrum of one resonance of TE and TM polarization mode for (a) Disk and (b) Donut device under +60 V (blue), 0 V (black), and -60 V (red). The x axis represents wavelength shifts relative to resonant wavelength $\lambda_0$ ($\sim$1550 nm) of each mode. The resonant wavelength decreases for the Disk, while it increases for the Donut under +60 V. The tuning direction reverses for opposite voltages, demonstrating bi-directional tuning. (c-d) Dependence of resonant wavelength detune on voltages for the Disk and Donut devices respectively. Experimental results (squares) show high linearity for both TE (pink) and TM (cyan) modes, with $R^2 > 99\%$ linear fitting (straight lines). The two actuator designs show different tuning directions and tuning ranges. (e-f) Influence of piezoelectric actuation on optical quality factor Q for TE and TM modes of both designs, which verifies that the actuation will not influence the optical Q.

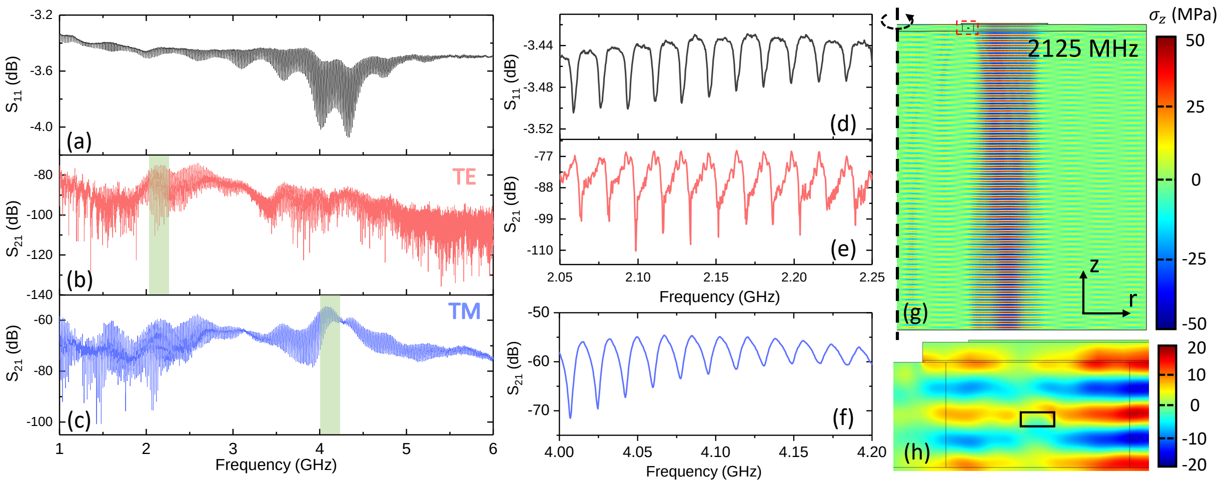


**Supplementary Figure 10:** The same $S_{11}$ and $S_{21}$ measurements on the Donut device in GHz range. (a) Electromechanical $S_{11}$ spectrum from 1 to 6 GHz. Optomechanical $S_{21}$ responses of (b) TE and (c) TM modes demonstrate effective stress-optical modulation spanning a broad range of microwave frequencies. The Donut device shows similar results as reported for the Disk device in the main text. (d) and (e) show the zoom-in of $S_{11}$ and $S_{21}$ responses of TE mode within the window (green shaded area) around 2 GHz in (b), while (f) shows the zoom-in of TM mode's $S_{21}$ response around 4 GHz in (c). (g) Numerical simulation of $\sigma_z$ distribution for one typical acoustic resonant mode at 2.125 GHz, with a zoom-in around the optical waveguide (red box) shown in (h).

distribution, envelope of resonances, and signal to noise ratio. In the zoom-in around 2 GHz in Fig. 10(e), there are multiple peaks inside each resonance due to existence of higher order acoustic modes. Numerical simulations of one of the fundamental modes at 2.125 GHz is shown in Fig. 10(g), and the zoom-in around waveguide is in Fig. 10(h). From the mode distribution, we can see the acoustic wave is effectively excited and confined under the actuator vertically with little diffraction angle. The good mode confinement guarantees low cross-talk between closely placed actuators.

## Supplementary Note 3: Electromechanical cross-talk between adjacent actuators

As mentioned in the previous section, the high confinement of the acoustic mode beneath the actuator guarantees low cross-talk between adjacent actuators. To demonstrate this, three actuators are closely placed on the same optical ring resonator, which cover the whole ring in an interval of 120°, as shown in Fig. 11(a). The one port reflection parameter $S_{11}$ of actuator 1 is first measured as illustrated in Fig. 11(b). The difference from the $S_{11}$ shown in the main text is caused by the thicker Si substrate (500 μm) and thicker $SiO_2$ cladding (7 μm). These lead to smaller FSR and period of the envelope.

More importantly, the cross-talk is measured by performing a two-port electromechanical

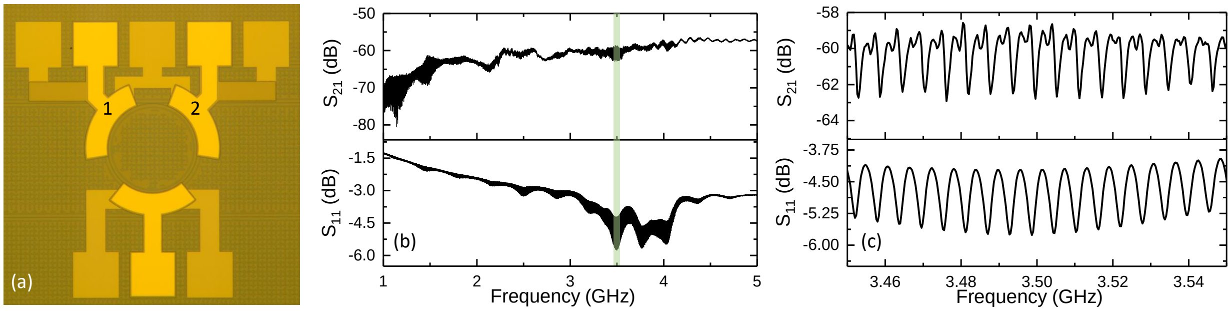


**Supplementary Figure 11:** Demonstration of low electromechanical cross-talk between adjacent actuators. (a) Optical microscope image of the device with three closely placed actuators. (b) (top) Two port electromechanical $S_{21}$ measurement by driving actuator 1 and sensing from actuator 2 as labeled in (a). (bottom) $S_{11}$ reflection for device 1. The cross-talk between the two devices is as low as -60 dB which guarantees compact integration. (c) Zoom-in of the measured $S_{21}$ and $S_{11}$ responses in the green shaded region in (b).

$S_{21}$ measurement, where we drive actuator 1 and sense the electrical signal out from the adjacent actuator 2. The cross-talk mainly comes from the leaking of acoustic waves from actuator 1 which can be sensed out by actuator 2 via the piezoelectric effect. The leakage of electric field will also be sensed by device 2 and cause cross-talk between electrical signals of device 1 and 2. As demonstrated in Fig. 11(b), $S_{21}$ as low as -60 dB of cross-talk is achieved, which illustrates the electrical and mechanical isolation between the two adjacent devices. This low cross-talk enables us to fabricate several actuators on the same optical ring, which may realize optical isolation through spatial-temporal modulation [7,8], or dispersion engineering of $Si_3N_4$ microring resonator [9] by engineering stress distribution.

## Supplementary Note 4: High Bandwidth Modulation

For applications where a wide bandwidth is required, the acoustic modes should be effectively damped or even eliminated. To do this, the backside surface of the Si substrate is first isotropically etched by $XeF_2$ in a Xactix Xenon Difluroide E1 system, with the top surface protected by the photoresist AZ1518. The roughing will diffract acoustic waves to random directions and thus weaken constructive interference. Next, as suggested by previous work[10], a layer of polyurethane (PU) epoxy (Permabond PT326) mixed with 3 μm nickle powder (GoodFellow) is pasted at the bottom, which can damp acoustic vibration at the boundaries and absorb acoustic energy, as shown in Fig. 12(a). After post-processing the fabricated device, its electromechanical and optomechanical performances are recorded in Fig. 12(b) and (c), respectively. To increase the signal to noise ratio of $S_{21}$ at high frequencies, the RF signal from the VNA is amplified (+30dB) before being applied to the actuator. From the $S_{11}$ response, one can see that nearly all resonances from the Si substrate cavity are diminished. However, the smoothly varying envelope from resonances in $SiO_2$ and AlN cavities still exists, since they remain unaffected by the post-processes. The

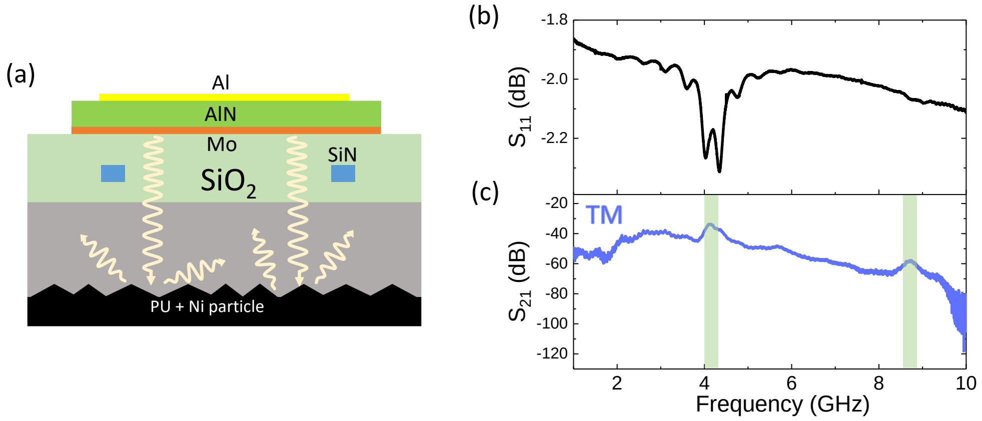


**Supplementary Figure 12:** (a) Cross-section of the device after roughing and then pasting polyurethane (PU) epoxy (mixed with 3 μm Nickle powders) on the backside of the Si substrate. (b) $S_{11}$ and (c) $S_{21}$ of the TM mode after weakening acoustic resonances. The VNA responses become smoother with only wide range envelope variation. Enabled only by AlN fundamental and second harmonic resonances (green shaded regions), broadband modulation can be achieved with 3 dB bandwidth of 250 MHz for each.

$S_{21}$ measurement also demonstrates smoothing of the modulation spectrum, but with broad range variations. Some exceptionally small resonances are still visible below 2.5 GHz in $S_{21}$, because the acoustic wavelength at low frequencies is comparable or larger than the Si roughing scale ($\sim$5 μm). It's worth noting here that the optical measurement is more sensitive than its electrical counterpart due to high optical Q and its signal to noise ratio. The fast roll-off of $S_{21}$ starting around 9.5 GHz is mainly limited by the optical quality factor of the TM mode, and the actual acousto-optic interaction may potentially extend to frequencies beyond 10 GHz.

The fundamental resonance from the AlN thin film enhances the electromechanical conversion efficiency and thus optical modulation around 4.13 GHz, and thanks to its low mechanical Q, we observe high bandwidth modulation with a 3 dB bandwidth of 250 MHz, as shown in Fig. 12(c). The second harmonic resonance of AlN cavity is also found in $S_{21}$ around 8.7 GHz, here with a 260 MHz bandwidth. These wide bands of modulation can potentially be used to connect supperconducting circuits with optical interfaces for low-loss quantum information communication [11]. Of note, the positions of these bands can be engineered by modifying the AlN and $SiO_2$ film thickness for specific applications. For future works, dedicated design and patterning of the bottom surface can be done for better suppression of acoustic resonances from Si cavity. To further increase modulation bandwidth, more effort is needed to eliminate resonances from $SiO_2$ and AlN layers, which can be realized by matching mechanical impedance at interfaces, such as using an acoustic anti-reflection layer [12], or through phononic crystal band gap engineering to suppress undesired acoustic modes [13].

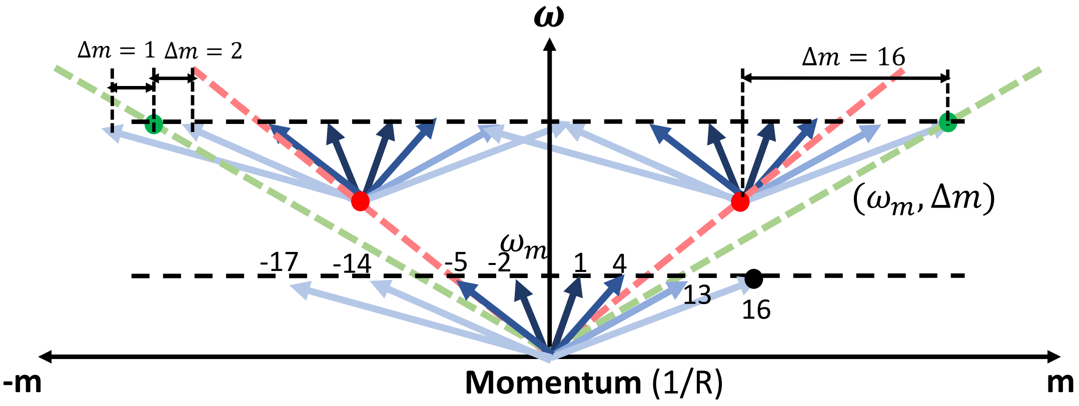


**Supplementary Figure 13:** Schematic for showing the phase matching condition for the two optical modes in the experiment. The azimuthal order difference between the two optical modes in the experiment is 16 which requires an effective modulation wave with azimuthal order of 16. Due to the discrete nature of the modulation regions, it contains multiple components with different azimuthal orders (the order of each is as labeled), one of which can be used to satisfy the phase matching condition. The color of each arrow denotes the relative strength of each component with darker with higher intensity. The momentum axis is represented by the azimuthal order in the unit of 1/R where R is the radius of the microring. Note this is just for schematic illustration with frequency and momentum not to the exact scale.

## Supplementary Note 5: Phase matching condition for $Si_3N_4$ ring isolator

**Effect due to the discrete modulation regions.** The energy conservation can be easily satisfied by driving the actuator at the frequency spacing of the two optical modes. Let's see how the phase matching (or momentum conservation) can be satisfied in the experiment. By comparing with the numerical finite element simulation (COMSOL), the azimuthal order difference between the two TE and TM mode is found to be 16. According to the resonant condition for both optical and mechanical cavity, the momentum is linked to the azimuthal orders as:

$$k_a = \frac{2\pi}{\lambda_a} = \frac{m_a}{R} \tag{38}$$

$$k_b = \frac{2\pi}{\lambda_b} = \frac{m_b}{R} \tag{39}$$

$$k_m = \frac{2\pi}{\lambda_m} = \frac{m_m}{R} \tag{40}$$

where $k_a$ ($m_a$), $k_b$ ($m_b$), and $k_m$ ($m_m$) are the momentum (azimuthal order) for optical mode $a$ and $b$ and the modulation wave. $R$ is the radius of the microring. As shown in Fig.13, an effective wave with azimuthal order of 16 should be excited for efficient scattering between the two modes. Due to the discrete nature of the modulation scheme, it excites many different Fourier components in

space with different azimuthal order[14]:

$$\sum_{n=-\infty}^{+\infty} \frac{\sin[(1+3n)\pi/3]}{(1+3n)\pi/3} \cos[\omega_m t - (1+3n)\phi] = 0.83\cos[\omega_m t - \phi] + 0.4\cos[\omega_m t + 2\phi] - 0.2\cos[\omega_m t - 4\phi] + \cdots - 0.0517\cos[\omega_m t - 16\phi] - 0.0486\cos[\omega_m t + 17\phi] \quad (41)$$

where $\phi$ is the azimuthal rotation angle, $\omega_m$ is the modulation frequency, '3' comes from the use of three actuators. We can see the component with 16 azimuthal order satisfies the momentum difference between the two optical modes. One important aspect of discrete modulation is the coexistence of effective waves in the backward direction. However, as shown in Fig.13, mode 16 only exists in the forward direction, whereas mode -17 exists in the other direction which is the closest mode with momentum mismatch of $\Delta m = 1$.

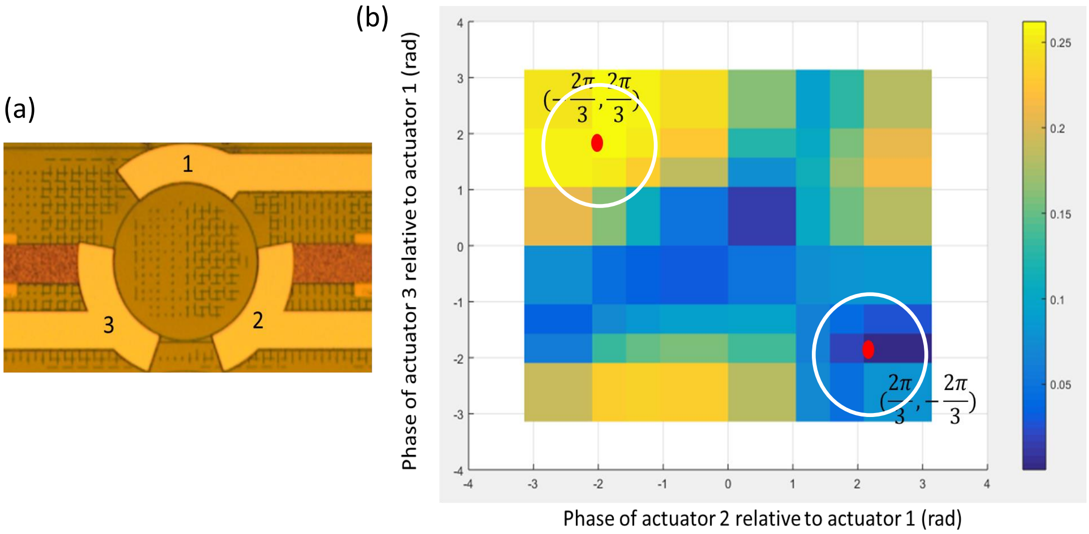


**Supplementary Figure 14:** (a) Optical microscope of the device with each actuator labeled with a referencing number. (b) Numerical simulation of the intensity of the converted sideband for various relative phases between the three actuators. Over a relatively broad RF phase variation (as labeled in the white circle) there is an observable nonreciprocity.

From the above analysis, we found it would be desirable to decrease the azimuthal order difference between TE and TM modes for improving the coupling efficiency and isolation. This can be seen in two ways. First, more modulation energy will be dropped into effective waves with smaller azimuthal order. For example, 69% (= $0.83^2$) energy is in mode 1. Thus, ideally we can optimize the waveguide design to have the optical mode difference to be 1. Second, the energy in the undesired backward wave with close mode order will be smaller. For example, mode -2 has energy of 16% (= $0.4^2$) which is much smaller than the energy in mode 1. Therefore, the device performance can be further improved by engineering the optical cavity to support TE and TM modes with order difference of 1.

**Sensitivity to the RF phase deviation from the ideal case.** As a practical consideration, it would be interesting to see how sensitive the nonreciprocity is relative to the deviation of RF phases from the ideal case. This is done by conducting numerical FDFD simulations[15] as shown in Fig.14. The converted light intensity is used to represent the coupling strength between the two optical modes. The phases of actuator 2 and 3 relative to actuator 1 is swept from $-\pi$ to $\pi$. To correctly read the figure we should note that reversing the input light direction is equivalent to reversing the sign of RF phase differences. From the figure we can find for phase configuration $(-\frac{2\pi}{3}, \frac{2\pi}{3})$ and its reverse case $(\frac{2\pi}{3}, -\frac{2\pi}{3})$, there is the maximum nonreciprocity contrast. However, the nonreciprocity contrast decreases gradually as the phases deviate from the ideal case, which suggests there is a wiggle room for RF phase tolerance, and no strict phase setting is required in order to observe nonreciprocity.

## References


1. Tirado, J. A. V. *Bulk acoustic wave resonators and their application to microwave devices*. Ph.D. thesis, Universitat Autònoma de Barcelona (2010).

2. Zhang, Y., Wang, Z. & Cheeke, J. D. N. Resonant spectrum method to characterize piezoelectric films in composite resonators. *ieee transactions on ultrasonics, ferroelectrics, and frequency control* **50**, 321–333 (2003).

3. Chen, Q. & Wang, Q.-m. Characterization of mechanical and piezoelectric properties of the aln thin film in a composite resonator stuctrue. In *2006 IEEE International Frequency Control Symposium and Exposition*, 104–110 (IEEE, 2006).

4. Tadesse, S. A. & Li, M. Sub-optical wavelength acoustic wave modulation of integrated photonic resonators at microwave frequencies. *Nature communications* **5**, 5402 (2014).

5. Huang, M. Stress effects on the performance of optical waveguides. *International Journal of Solids and Structures* **40**, 1615–1632 (2003).

6. Xu, D.-X. Polarization control in silicon photonic waveguide components using cladding stress engineering. In *Silicon Photonics II*, 31–70 (Springer, 2011).

7. Shi, Y., Lin, Q., Minkov, M. & Fan, S. Nonreciprocal optical dissipation based on direction-dependent rabi splitting. *IEEE Journal of Selected Topics in Quantum Electronics* **24**, 1–7 (2018).

8. Sohn, D. B., Kim, S. & Bahl, G. Time-reversal symmetry breaking with acoustic pumping of nanophotonic circuits. *Nature Photonics* **12**, 91 (2018).

9. Yao, B. *et al.* Gate-tunable frequency combs in graphene–nitride microresonators. *Nature* **558**, 410 (2018).

10. Pinrod, V., Davaji, B. & Lal, A. Coexisting surface and bulk gyroscopic effects. In *2018 IEEE International Ultrasonics Symposium (IUS)*, 1–4 (IEEE, 2018).

11. Kurizki, G. *et al.* Quantum technologies with hybrid systems. *Proceedings of the National Academy of Sciences* **112**, 3866–3873 (2015).

12. Zhu, X., Wei, Q., Cheng, Y., Wu, D. & Liu, X. Perfect monochromatic acoustic anti-reflection: A first-principles study. *Journal of Applied Physics* **121**, 094504 (2017).

13. Lu, M.-H., Feng, L. & Chen, Y.-F. Phononic crystals and acoustic metamaterials. *Materials today* **12**, 34–42 (2009).

14. Sounas, D. L. & Alu, A. Angular-momentum-biased nanorings to realize magnetic-free integrated optical isolation. *ACS photonics* **1**, 198–204 (2014).

15. Shi, Y. Two-dimensional finite-difference frequency-domain (fdfd) programs (2018). URL https://github.com/YuJerryShi/fdfd_suite.
