# Supplementary material for: Hybrid integrated photonics using bulk acoustic resonators
Source: Nat Commun. 2020 Jun 17;11:3073. doi: 10.1038/s41467-020-16812-6 (PMC7299988; doi:10.1038/s41467-020-16812-6)
Supplement: Supplementary file 2 — Peer Review File [file 41467_2020_16812_MOESM2_ESM.pdf]

Editorial Note: This manuscript has been previously reviewed at another journal that is not operating a transparent peer review scheme. This document only contains reviewer comments and rebuttal letters for versions considered at Nature Communications.
